# A systematic approach to normalization in probabilistic models

**DOI:** 10.1007/s10791-018-9334-1

**Published:** 2018-06-30

**Authors:** Aldo Lipani, Thomas Roelleke, Mihai Lupu, Allan Hanbury

**Affiliations:** 1 TU Wien, Vienna, Austria; 2 Queen Mary University of London, London, UK; 3 Research Studios Austria, Vienna, Austria; 4 Complexity Science Hub, Vienna, Austria

**Keywords:** Verboseness hypothesis, TF normalization, Smoothing

## Abstract

Every information retrieval (IR) model embeds in its scoring function a form of term frequency (TF) quantification. The contribution of the term frequency is determined by the properties of the function of the chosen TF quantification, and by its TF normalization. The first defines how independent the occurrences of multiple terms are, while the second acts on mitigating the a priori probability of having a high term frequency in a document (estimation usually based on the document length). New test collections, coming from different domains (e.g. medical, legal), give evidence that not only document length, but in addition, verboseness of documents should be explicitly considered. Therefore we propose and investigate a systematic combination of document verboseness and length. To theoretically justify the combination, we show the duality between document verboseness and length. In addition, we investigate the duality between verboseness and other components of IR models. We test these new TF normalizations on four suitable test collections. We do this on a well defined spectrum of TF quantifications. Finally, based on the theoretical and experimental observations, we show how the two components of this new normalization, document verboseness and length, interact with each other. Our experiments demonstrate that the new models never underperform existing models, while sometimes introducing statistically significantly better results, at no additional computational cost.

## Introduction

The development of retrieval models is one of the key aspects of research in information retrieval (IR). The IR models arise from experimental observations about the use of the language, predominantly on collections of documents primarily composed of news corpora. Today, with the almost total digitization of most text produced, it is clear that the textual documents are not just news and that different collections require different approaches (Hanbury and Lupu 2013). Consequently, the field has been driven to deal with different kinds of information types, demonstrated by the creation of new and more domain specific initiatives in the main IR evaluation campaigns: TREC, NTCIR, CLEF, and FIRE. Now, thanks to the observations made in the context of these evaluation campaigns, we are able to revisit some of the original assumptions and extend the models to integrate other collection statistics that reflect the different use of the language in different domains.

Every IR model boils down to a scoring function in which we can distinguish a component that increases with the number of occurrences of a term in a document (a term frequency component, ${\text {TF}}$) and a component that decreases with the commonality of a term (an inverse document frequency component, ${\text {IDF}}$). In this paper we focus on the ${\text {TF}}$ component. Its normalization, first introduced by Robertson et al. (1994) for BM25, and then generalized by Singhal et al. (1996) for a generic model, consists in adjusting the within-document term frequency ($\textit{tf}_d$) based on the ratio between the document length ($l_d$) and its expectation ($\mathrm {E}_{\mathcal {D}}[l_d]$), called pivoted document length normalization. The work of Singhal et al. is motivated by the experimental observation that the length pattern of the retrieved documents should match the pattern of the relevant documents. Robertson et al. justify this normalization, later declared as ‘soft’ for the mitigation effect provided by the division by the mean, by introducing two contrasting hypotheses (Robertson and Zaragoza 2009), named *verboseness* and *multi-topicality*: (a) the verboseness hypothesis states that some authors need more words to explain something that could have been explained with fewer; (b) the multi-topicality hypothesis states that the reason why more words are required is because the author has covered more ground. While the first hypothesis suggests a document should be normalized by its length, the second suggests the contrary.

Recently, Lipani et al. (2015) have brought back to the attention of the IR community this discussion, pointing out that another collection statistic could be embedded in the ${\text {TF}}$ normalization of BM25. This new statistic measures a kind of verboseness, the repetitiveness of terms in a document, and leads to the achievement of performance better than the standard BM25.

In this paper we address this new observation from the perspective of the established models, and provide a new, general theory. Before doing that, a few general observations are in order.

Retrieval models combine various parameters into a score reflecting the degree to which a document implies a query. The common parameters and rationales are:

$$\textit{tf}_d$$
within-document term frequency; frequent is good
$$P_D(t|c)$$
document-based term prob. (aka ${\text {IDF}}(t,c) = -\log (P_D(t|c))$); rare is good
*P*(*t*|*c*): occurrence-based term probability (LM mixture)
$$l_d$$
document length; to promote short documents

where *c* is a collection of documents, *d* is a document, and *t* is a term. We claim that there are other properties of documents and terms that are important but under-represented, namely verboseness and the previously introduced burstiness (Roelleke 2013). In this paper we will focus primarily on verboseness, but we will also make some observations on burstiness and its relation with ${\text {IDF}}$. However, before starting, we introduce the notation used.

### Notation

The basic symbols and sets are given in the following table. The notation is based on the proposal made by Roelleke (2013). However, unlike Roelleke, given that here we will not theoretically analyze different collections, we will generally drop the collection *c* index where convenient and not ambiguous.

**Table:** 

$\mathcal {T}$	set of terms in the collection
$\mathcal {D}$	set of documents in the collection
*t*	a term $t\in \mathcal {T}$
*d*	a document $d\in \mathcal {D}$
$|\mathcal {T}|$	number of terms
$|\mathcal {D}|$	number of documents
$l_c$	length of collection (number of term occurrences)

Based on the basic symbols, we define frequencies. Term frequencies, document frequencies, average term frequencies are ambiguous notions. It is important to clarify exactly what symbols mean.

**Table:** 

$l_t$	number of occurrences of the term *t* in the collection, here also called term length (aka collection frequency)
$\mathcal {D}_t$	set of documents where *t* occurs
$\mathcal {T}_d$	set of terms in *d*
$|\mathcal {D}_t|$	number of documents where *t* occurs (aka document frequency, ${\text {df}}(t)$)
$|\mathcal {T}_d|$	number of distinct terms in *d*
$l_d$	length of document *d* (number of term occurrences, note $l_d \ge |\mathcal {T}_d|$)

Next, we define the four averages important for this paper. The first two combine in a systematic way the symbols of the previous table.

**Table:** 

$\mathrm {E}_{\mathcal {D}_t}[\textit{tf}_d] = l_t/|\mathcal {D}_t|$	average frequency of term *t* in the documents in which the term occurs
$\mathrm {E}_{\mathcal {T}_d}[\textit{tf}_d]=l_d/|\mathcal {T}_d|$	average term frequency of terms that occur in document *d*
$\bar{l}_d := \mathrm {E}_{\mathcal {D}}[l_d] = l_c/|\mathcal {D}|$	average document length
$\bar{l}_t := \mathrm {E}_{\mathcal {T}}[l_t] = l_c/|\mathcal {T}|$	average term length

Note that there are two notions regarding “average term frequency”, $\mathrm {E}_{\mathcal {D}_t}[\textit{tf}_d]$ and $\mathrm {E}_{\mathcal {T}_d}[\textit{tf}_d]$. In the first case the average is performed fixing *t* and averaging across the documents $\mathcal {D}_t$ containing *t*, and in the second case the average is performed fixing *d* and averaging across the terms $\mathcal {T}_d$ contained therein.

Finally, we introduce the probabilities used in this paper.

**Table:** 

$P(t)=P_L(t)=l_t/l_c$	location based probability of $t\in \mathcal {T}$
$P(d)=P_L(d)=l_d/l_c$	location based probability of $d\in \mathcal {D}$
$P_D(t)=|\mathcal {D}_t|/|\mathcal {D}|$	document based probability of $t\in \mathcal {T}$
$P_T(d)=|\mathcal {T}_d|/|\mathcal {T}|$	term based probability of $d\in \mathcal {D}$

As can be seen, in this paper, when mentioning probability (*P*) with no index we refer to the probability based on locations, i.e. the probability defined on the sample space of term occurrences.

### Motivations

In this section we formally introduce the document verboseness and term burstiness. We then motivate their investigation in IR models.

*Verboseness* is reflected by the ratio $l_d/|\mathcal {T}_d|$: the document length divided by the number of (distinct) terms in the document. The ratio corresponds to *the average *$\textit{tf}_d$* (over all terms) in document* *d*:

1 $$\begin{aligned} v_d:=\mathrm {E}_{\mathcal {T}_d}[\textit{tf}_d] = \frac{l_d}{|\mathcal {T}_d|} \end{aligned}$$

A document is verbose if few terms are repeated many times; its domain is $[1, l_d]$, 1 for non-verbose (no term occurs more then once), and $l_d$ for maximally verbose (one term is repeated $l_d$ times).

Intuitively, the more verbose (repetitive) a document is, the higher is the chance to find a high $\textit{tf}_d$. In other words, a document has a high score just because words are repeated (e.g. spamming), and therefore, one wants to demote verbose documents in the ranking.

*Burstiness* is reflected by the ratio $l_t/|\mathcal {D}_t|$, that is the length of the term in the collection *c* (or number of occurrences of the term in *c*) divided by the number of the collection’s documents where the term *t* occurs (aka document frequency). The ratio corresponds to *the average *$\textit{tf}_d$* (over the number of documents where the term* *t occurs) in collection* *c*:

2 $$\begin{aligned} b_t:=\mathrm {E}_{\mathcal {D}_t}[\textit{tf}_d] = \frac{l_t}{|\mathcal {D}_t|} \end{aligned}$$

A term is bursty if it occurs in few documents many times; its domain is $[1, l_t]$, 1 for a non-bursty term (it occurs only once in each document where it is present), $l_t$ for maximally bursty (all the occurrences are only in one document).

Intuitively, the more bursty a term is, the higher is the chance to find a high $\textit{tf}_d$. In other words, a bursty term occurs in fewer documents than a non-bursty (a normal) term, and therefore, one wants to promote documents containing bursty terms.

Instead of verboseness and burstiness, scoring functions most often use normalization of the $\textit{tf}_d$ based on the document length $l_d$ (e.g. in the TF component of BM25 and in some versions of TF-IDF) .

The contribution of the *document length* is smoothed by its average, that corresponds to *the average *$l_d$* (over all the documents) in collection* *c*:

3 $$\begin{aligned} \text {avgdl}(c) = \mathrm {E}_{\mathcal {D}}[l_d] = \frac{l_c}{|\mathcal {D}|} \end{aligned}$$

This is then used to calculate the pivoted document length (pivotization indicated in the paper with a hat) as follows:

4 $$\begin{aligned} \hat{l}_d := \frac{l_d}{\mathrm {E}_{\mathcal {D}}[l_d]} \end{aligned}$$

The $\hat{l}_d$ is greater than 1 for relatively long documents (greater than the average document length), and smaller than 1 for short documents (lower than the average document length).

It is surprising that IR models are keen to capture the $\hat{l}_d$, but seem to hide away verboseness and burstiness, i.e. there is no parameter explicitly associated with these properties. However we observe that some IR models implicitly use these normalizations.

We investigate which IR models capture verboseness and burstiness, and how the parameters can be made explicit or added. Motivated by the work of Lipani et al. (2015), we formally justify verboseness from its duality with the document length normalization. As a supportive case we also present its duality with the concept of burstiness (Roelleke 2013), and term length (aka collection frequency).

### Contributions and structure

The main contributions of this paper are: (1) The inclusion of document verboseness as an explicit parameter in TF quantifications, showing that verboseness is to be viewed in a similar way as the document length in the TF normalizations; (2) An extensive set of experiments capturing a well-defined spectrum of TF quantifications, whose results for log-based and BM25-based TF quantifications deliver a significant contribution to insights into the effect of TF quantifications, even beyond the TF normalization variants; (3) Theoretical justifications for the way document verboseness and length are combined, considering the dualities between verboseness and other parameters (including the burstiness of terms).

The remainder of the paper is structured as follows: in Sect. 2 we present the background. In Sect. 3, the main contribution of the paper, namely combining document verboseness and length into the normalization parameter $K_d$ of the TF quantification, is presented. We next review in Sect. 4 the probabilistic foundations of IR models. This highlights the role of parameters such as verboseness, burstiness and document length, and the theoretical justification of $\text {TF}_{\text {BM25}}$-IDF. In Sect. 5, we report the experimental setup and results, followed by Sect. 6 dedicated to the discussion of the results. Section 7 concludes the paper.

## Background

The discussion about the TF normalization was initiated by Robertson and Zaragoza (2009), introducing the two hypotheses: verboseness and multi-topicality and then followed by the work of Singhal et al. (1996) where the document length pivotization is justified experimentally. Not much work has been done on the multi-topicality hypothesis, but some for the verboseness hypothesis. However, the problem of how to weight terms dates back further, to the work of Salton and Buckley (1988). Na et al. (2008) introduce the concept of repetitiveness to derive a smoothing method for Language Modeling, showing an improvement with respect to other smoothing methods.

Following other work on the TF normalization issues, He and Ounis (2005a) apply the Dirichlet priors to the TF normalization following the idea of Amati and Van Rijsbergen (2002), and test it on different test collections (He and Ounis 2003, 2005b). Lv and Zhai pointed out that the TF quantification based on document length excessively penalizes very long documents due to its lower bound, a problem mitigated by leveraging the TF normalization by adding a constant (Lv and Zhai 2011b). They also pointed out that in case of BM25 it can be mitigated by adding a constant to the TF normalization (Lv and Zhai 2011c). Rousseau and Vazirgiannis (2013) generalized the previously mentioned TF normalizations through functional composition. Lv and Zhai (2011a) estimate dynamically the parameter $k_1$ of BM25, based on a proposed information gain measure.

Lipani et al. (2015) introduce a new variant of BM25, called BM25VA that explicitly incorporates verboseness. This is the main work that motivates this paper. The verboseness is defined as in Eq. (1), and pivoted as $v_d/\mathrm {E}_{\mathcal {D}}[v_d]$. Verboseness is then added to the $\text {TF}_{\text {BM25}}$, linearly combining the two contributions through the parameter *b*, as follows:

5 $$\begin{aligned} K_d := k_1 \cdot \left[ (1-b) \cdot \hat{v}_d + b \cdot \hat{l}_d \right] \end{aligned}$$

In this work, it is heuristically shown that the parameter *b* is inversely proportional to a statistic of the collection, the average collection verboseness $\mathrm {E}_{\mathcal {D}}[v_d]$, and that it can be predicted without statistically damaging the performance of the trained BM25.

Another way of approaching the length normalization issue is to consider retrieval of the the individual passages (Robertson and Walker 1999). However, this use of passages to address length normalization is theoretically unjustified and introduces a series of decision points (size and nature of passages) that are not the focus of this current study.

## TF normalisations

Before getting into the details of the duality between document verboseness and length, it is necessary to formally define the current pivotization of document length and introduce the pivotization of verboseness. To do this we start from the foundation of every IR model: the document-term matrix $A \in \mathbb {N}^{|\mathcal {D}| \times |\mathcal {T}|}$, in which each element is a $\textit{tf}_d$ indicated here by $a_{d,t}$ for convenience of the notation. For any given matrix, we can define two ways to sum the elements of this matrix; one that fixes a column (a term *t*) and sums over the rows (the $|\mathcal {D}|$ documents) and one that fixes a row (a document *d*) and sums over the columns (the $|\mathcal {T}|$ terms). Doing this we calculate two lengths: the length of a term^1^ and the length of a document, as follows:

6 $$\begin{aligned} \sum _{d \in \mathcal {D}} a_{d,t} = l_t \qquad \sum _{t \in \mathcal {T}} a_{d,t} = l_d \end{aligned}$$

Now, if we want to compute the average of the values on each row or column, we have to divide the sums obtained above by a *value*. For this *value* we actually have two options: the number of columns or rows, and the number of non-zero elements in the columns or rows. The first is what we would call the *average*, and the second the *elite average*. To give an intuition, think of the question *“What is the average number of Ferraris owned by a person?”*. This question has two answers: we can divide the total number of Ferraris (the sum of the elements on a row/column) by the total number of people on the planet (the number of columns/rows); or, we can consider only those people that have at least one Ferrari and then divide the number of Ferraris by the size of this set of people. The first one is the common average, while the second, obviously, is the *elite* average.

**Fig. 1:**
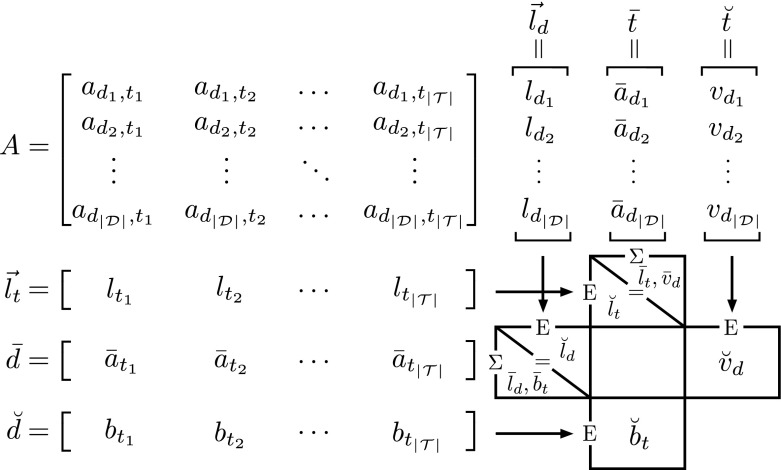
The graphical rapresentation of the calculation performend in Sect. 3. On the top left corner we show the matrix $A \in \mathbb {N}^{|\mathcal {D}| \times |\mathcal {T}|}$. To the right of *A* we show three vectors, $\vec {l}_d$, $\bar{t}$, and $\breve{t}$, obtained by performing a summation, an average and an elite average of the columns of *A*. On the bottom of *A* we show three vectors $\vec {l}_t$, $\bar{d}$, and $\breve{d}$, obtained by performing a summation, an average and an elite average of the rows of *A*. On the bottom right corner we show the set of collection statistics, calculated using these six vectors, obtained by performing a summation (when indicated by a $\Sigma$) or an average (when indicated by an $\text {E}$) of the values of the vector on the top of the operator sign if the operator sign is shown on the top side of the block, or on the left of the operator sign if the operator sign is shown on the left side of the block. All the collection statistics appearing in the same block are equivalent, e.g. $\breve{l}_t = \bar{l}_t = \bar{v}_d$

Returning to our document-term matrix, we will denote by a bar ($\bar{a}$) a common average and by a breve $(\breve{a}$) an elite average:

7 $$\begin{aligned} \begin{aligned} \bar{a}_t&=\frac{1}{|\mathcal {D}|}\sum _{d \in \mathcal {D}} a_{d,t} = \frac{l_t}{|\mathcal {D}|} \\ \breve{a}_t&=\frac{1}{|\{a_{d,t} : a_{d,t}\ne 0\}|}\sum _{d \in \mathcal {D}} a_{d,t} = \frac{1}{|\mathcal {D}_t|}\sum _{d \in \mathcal {D}} a_{d,t} = \frac{l_t}{|\mathcal {D}_t|} = b_t \\ \bar{a}_d&=\frac{1}{|\mathcal {T}|}\sum _{t \in \mathcal {T}} a_{d,t} = \frac{l_d}{|\mathcal {T}|}\\ \breve{a}_d&=\frac{1}{|\{a_{d,t} : a_{d,t}\ne 0\}|}\sum _{t \in \mathcal {T}} a_{d,t} = \frac{1}{|\mathcal {T}_d|}\sum _{t \in \mathcal {T}} a_{d,t} = \frac{l_d}{|\mathcal {T}_d|} = v_d\\ \end{aligned} \end{aligned}$$

in which we observe that the two elite averages just defined $\breve{a}_t$ and $\breve{a}_d$ correspond to the burstiness $b_t$ as defined in Eq. (2) and the verboseness $v_d$ as defined in Eq. (1).

Considering the remaining elements, $\bar{a}_t$, $\breve{a}_t$, $\bar{a}_d$ and $\breve{a}_d$, we can think of them as defining an average document $\bar{d} = [\bar{a}_{t_1}\,\ldots \,\bar{a}_{t_{|\mathcal {T}|}}]$, an elite average document $\breve{d} = [\breve{a}_{t_1}\,\ldots \,\breve{a}_{t_{|\mathcal {T}|}}]$, an average term $\bar{t} = [\bar{a}_{d_1}\,\ldots \,\bar{a}_{d_{|\mathcal {D}|}}]$, and an elite average term $\breve{t} = [\breve{a}_{d_1}\,\ldots \,\breve{a}_{d_{|\mathcal {D}|}}]$. Moreover, we observe also that the elite average document is equal to $\breve{d} = [b_{t_1}\,\ldots \,b_{t_{|\mathcal {T}|}}]$ and the elite average term is equal to $\breve{t} = [v_{d_1}\,\ldots \,v_{d_{|\mathcal {D}|}}]$.

So, now, for each row *d* and for each column *t* we have a sum, an average, and an elite average. To obtain a collection-level statistic, we have to aggregate again, calculating sums and averages (common and elite averages are identical now, because all rows and all columns have a non-zero aggregated value).

Doing so, we observe that

8 $$\begin{aligned} \breve{l}_d:=\frac{1}{|\mathcal {D}|}\sum _{d \in \mathcal {D}}l_d \qquad \bar{l}_d:= \sum _{t \in \mathcal {T}}\bar{a}_t = \frac{l_c}{|\mathcal {D}|} \qquad \breve{l}_d = \bar{l}_d \end{aligned}$$

i.e. the average document length $\bar{l}_d$ is equal to the sum of the elements of the average document $\bar{d}$.

However, the same observation is not valid for verboseness, because it is an elite average. Instead, we have two notations:

9 $$\begin{aligned} \breve{v}_d:=\frac{1}{|\mathcal {D}|}\sum _{d \in \mathcal {D}}v_d \qquad \bar{v}_d:=\sum _{d \in \mathcal {D}}\bar{a}_d=\frac{l_c}{|\mathcal {T}|} \qquad \breve{v}_d \ne \bar{v}_d \end{aligned}$$

A graphical representation of the calculations performed in this section is shown in Fig. 1.

### Duality: document verboseness and length

Recalling the definition of verboseness from Eq. (1), it is the average number of times a document’s term occurs within the document. To observe the duality of document verboseness, Eq. (3), let us first define the notation to identify the singleton of a document $d \in \mathcal {D}$ as $\mathcal {D}_d=\{d\}$ and the singleton of a term $t \in \mathcal {T}$ as $\mathcal {T}_t=\{t\}$. Obviously $|\mathcal {D}_d|=|\mathcal {T}_t|=1$ and therefore we can write $l_d=l_d/|\mathcal {D}_d|$. Let us now consider the pivoted verboseness and pivoted document length, using the two sets of values defined above: $\bar{l}_d=\breve{l}_d$, $\bar{v}_d$ and $\breve{v}_d$:

10 $$\begin{aligned} \ddot{l}_d= & {} \frac{l_d}{\bar{l}_d} = \frac{l_d/|\mathcal {D}_d|}{l_c/|\mathcal {D}|} \end{aligned}$$

11 $$\begin{aligned} \hat{l}_d= & {} \frac{l_d}{\breve{l}_d} = \frac{l_d/|\mathcal {D}_d|}{\mathrm {E}_{\mathcal {D}}[l_d/|\mathcal {D}_d|]} \end{aligned}$$

12 $$\begin{aligned} \ddot{v}_d= & {} \frac{v_d}{\bar{v}_d} = \frac{l_d/|\mathcal {T}_d|}{l_c/|\mathcal {T}|} \end{aligned}$$

13 $$\begin{aligned} \hat{v}_d= & {} \frac{v_d}{\breve{v}_d} = \frac{l_d/|\mathcal {T}_d|}{\mathrm {E}_{\mathcal {D}}[l_d/|\mathcal {T}_d|]} \end{aligned}$$

where we indicate the non-elite pivotization with a double dots and the elite pivotization with a hat. The duality is obtained substituting $\mathcal {D}\rightarrow \mathcal {T}$ to go from $l_d$ to $v_d$ or $\mathcal {T}\rightarrow \mathcal {D}$ to go from $v_d$ to $l_d$.

The pivoted verboseness of a document is with respect to the space of terms ($\mathcal {T}$), whereas the pivoted document length of a document is with respect to the space of documents ($\mathcal {D}$). One can also show the duality between document verboseness and length based on probabilistic expressions:

14 $$\begin{aligned} \ddot{l}_d=\frac{l_d}{\bar{l}_d} = \frac{P_L(d)}{P_D(d)}=\frac{l_d/l_c}{|\mathcal {D}_d|/|\mathcal {D}|} \end{aligned}$$

15 $$\begin{aligned} \hat{l}_d=\frac{l_d}{\breve{l}_d} = \frac{P_L(d)/P_D(d)}{\mathrm {E}_{\mathcal {D}}[P_L(d)/P_D(d)]} \end{aligned}$$

16 $$\begin{aligned} \ddot{v}_d=\frac{v_d}{\bar{v}_d} =\frac{P_L(d)}{P_T(d)}=\frac{l_d/l_c}{|\mathcal {T}_d|/|\mathcal {T}|} \end{aligned}$$

17 $$\begin{aligned} \hat{v}_d=\frac{v_d}{\breve{v}_d} = \frac{P_L(d)/P_T(d)}{\mathrm {E}_{\mathcal {D}}[P_L(d)/P_T(d)]} \end{aligned}$$

$P_L(d)$ is the location based probability of a document. Dividing this by the term based probability of *d*, $P_T(d)=|\mathcal {T}_d|/|\mathcal {T}|$ yields the pivoted verboseness. Dividing by the document based probability of *d*, $P_D(d)=|\mathcal {D}_d|/|\mathcal {D}|=1/|\mathcal {D}|$, yields the pivoted document length.

The dualities between average document verboseness and average document length justify the combination of parameters as formalized in the definition capturing the normalization variants of $K_d$:

#### **Definition 1**

(TF Normalisations $$K_d$$ **)**

- $\ddot{K}_d$: the non-elite normalization comprises the non-elite pivots $\ddot{l}_d$ and $\ddot{v}_d$.
- $\hat{K}_d$: the elite normalization comprises the elite pivots $\hat{l}_d$ and $\hat{v}_d$.
- The expression ${\text {pivdl}}$, pivoted document length, denotes one of the two:

$$\begin{aligned} {\text {pivdl}}=\left\{ \begin{array}{ll} \ddot{l}_d &{} \text {non-elite pivot}\\ \hat{l}_d &{} \text {elite pivot}\\ \end{array} \right. \end{aligned}$$

Analogously for $\text {pivdv}$, pivoted document verboseness.

Then, the pivotization components are defined for the disjunctive (linear) and conjunctive (product) combination of the pivots.

18 $$\begin{aligned} \text {comb}\_\text {piv}_{b, a,\vee }(d) := 1 - b + b \cdot \left[ (1 - a)\cdot {\text {pivdl}}+ a\cdot \text {pivdv}\right] \end{aligned}$$

19 $$\begin{aligned} \text {comb}\_\text {piv}_{b, a,\wedge }(d) := \left[ {\text {pivdl}}^{1 - a} \cdot \text {pivdv}^{a} \right] ^b \end{aligned}$$

where the two parameters *b* and *a* are both defined in [0, 1]. The parameter *b* controls the degree of normalization between full normalization (when $b=1$) and no normalization (when $b=0$), and the parameter *a* controls the balance between the contributions of ${\text {pivdl}}$ and $\text {pivdv}$. The combination of these pivots becomes part of the usual definition of the normalization parameter $K_d$.

20 $$\begin{aligned} K_d = k_1 \cdot \text {comb}\_\text {piv}(d) \end{aligned}$$

where the parameter $k_1$, which is defined in $]0, \infty [$, controls the power of the normalization.

It is worth pointing out now that for $b=0$, or $b=1$ and $a=\{0,1\}$ these two combinations are the same. In particular we should note that:

21 $$\begin{aligned} \text {comb}\_\text {piv}_{0,a,\wedge }(d) = \text {comb}\_\text {piv}_{0,a,\vee }(d) = 1 \end{aligned}$$

which is the “traditional” $K_d$, created ignoring both document verboseness and length ($b=0$).

To summarize, there are four variants of the pivotization factor $K_d$: non-elite disjunctive denoted as $\ddot{K}_{\vee }$, non-elite conjunctive denoted as $\ddot{K}_{\wedge }$, and the respective elite variants $\hat{K}_{\vee }$ and $\hat{K}_{\wedge }$. The experiments emphasize the analysis of the behavior of these four variants.

### Example of calculation of the pivotizations

The next example illustrates the arithmetic to compute the pivoted document verboseness and length.

#### *Example 1*

(Pivoted Document Verboseness and Length) Assume a document *d* with $l_d=300$ word occurrences, and $|\mathcal {T}_d|=150$ distinct words. The verboseness is:

$$\begin{aligned} v_d = \frac{l_d}{|\mathcal {T}_d|}=\frac{300}{150} = 2 \end{aligned}$$

Let the collection contain $l_c=10^7$ word occurrences, and $|\mathcal {T}|=10^5$ distinct words. The non-elite average document verboseness is 100, that is, in average, a term occurs $\bar{v}_d=100$.

The elite average verboseness is the average over the verboseness values of the documents. For example, let $\breve{v}_d=5/2$ be the elite verboseness.

The pivoted verboseness is the verboseness divided by the average verboseness, e.g. the non-elite average verboseness:

$$\begin{aligned} \ddot{v}_d = \frac{v_d}{\bar{v}_d} =\frac{2}{100} = \frac{1}{50} \end{aligned}$$

while the pivoted elite verboseness is the verboseness divided by the elite average verboseness:

$$\begin{aligned} \hat{v}_d = \frac{v_d}{\breve{v}_d} = \frac{2}{5/2} = \frac{4}{5} \end{aligned}$$

Regarding the document length, let $\bar{l}_d=400$ be the average document length. Then, the pivoted document length is:

$$\begin{aligned} \ddot{l}_d = \frac{l_d}{\bar{l}_d} = \frac{300}{400} = \frac{3}{4} \end{aligned}$$

Then we can combine the non-elite pivots, for example, in a disjunctive way:

$$\begin{aligned} \ddot{K}_{\vee ,d} = k_1\cdot \left\{ 1-b + b \cdot \left[ (1-a) \cdot \frac{3}{4} + a \cdot \frac{1}{50}\right] \right\} \end{aligned}$$

or, the elite pivots in a conjunctive way:

$$\begin{aligned} \hat{K}_{\wedge ,d} = k_1\cdot \left[ \left( \frac{3}{4}\right) ^a \left( \frac{4}{5}\right) ^{1-a}\right] ^b \end{aligned}$$

The other two variants, elite pivots combined in a disjunctive way ($\hat{K}_{\vee ,d}$), and non-elite pivots combined in a conjunctive way ($\ddot{K}_{\wedge ,d}$) are left to the reader.

### Other dualities

To strengthen the theoretical justifications, we explore two other dualities, namely the duality between document verboseness and term burstiness, and later in the section the duality between term burstiness and term length. Here, the definitions of the first couple:

22 $$\begin{aligned} \begin{aligned} \text {document verboseness: } v_d&:= l_d/|\mathcal {T}_d|\\ \text {term burstiness: } b_t&:= l_t/|\mathcal {D}_t| \end{aligned} \end{aligned}$$

The duality is obtained substituting $\mathcal {T}\rightarrow \mathcal {D}$ and $d \rightarrow t$ to go from $v_d$ to $b_t$ or $\mathcal {D}\rightarrow \mathcal {T}$ and $t \rightarrow d$ to go from $b_t$ to $v_d$. Verboseness is the average term frequency when considering the document length $l_d$ over the set $\mathcal {T}_d$ of terms that occur in the respective document. Burstiness is the average term frequency when considering the number of times the term occurs $l_t$ over the set $\mathcal {D}_t$ of documents in which the respective term occurs.

Furthermore, starting from burstiness and substituting $\mathcal {D}\rightarrow \mathcal {T}$, we observe another duality, between term length and burstiness:

23 $$\begin{aligned} \begin{aligned} \text {term burstiness: } b_t&:= l_t/|\mathcal {D}_t|\\ \text {term length: } l_t&:= l_t/|\mathcal {T}_t| \end{aligned} \end{aligned}$$

These dualities, based fundamentally on substitutions between the set of documents $\mathcal {D}$ and the set of terms $\mathcal {T}$, were briefly explored in the early 1990s, when Knaus et al. (1994), and Amati and Kerpedjiev (1992) talked about ITF (inverse term frequency) and IDF. IDF later generalized by Metzler (2008).

Whereas the IDF is applied for reasoning about the similarity between *documents*, the ITF is applied for reasoning about the similarity between *terms*. Viewing the ITF and IDF together, by looking at the denominator’s argument of the logarithms, shows that ITF is related to verboseness, and IDF is related to burstiness.

$$\begin{aligned} \text {ITF}(d,c):= & {} -\log \left( P_T(d|c)\right) \qquad \left( =\log \left( \frac{|\mathcal {T}|}{|\mathcal {T}_d|}\right) \right) \\ \text {IDF}(t,c):= & {} -\log \left( P_D(t|c)\right) \qquad \left( =\log \left( \frac{|\mathcal {D}|}{|\mathcal {D}_t|}\right) \right) \end{aligned}$$

Overall, the discussion supports the case to consider verboseness as a document-specific parameter, whereas traditional IR focuses on the pivoted document length only.

### Summary

This section justified the systematic combination of pivoted document length and pivoted verboseness, while placing them in the context of other dualities, involving burstiness and term length. Table 1 shows the list of all the explored dualities.

**Table 1:** List of all four dual properties

Document verboseness	$v_d := l_d/|\mathcal {T}_d|$
Document length	$l_d := l_d/|\mathcal {D}_d|$ (noting that $|\mathcal {D}_d|=1$)
Term burstiness	$b_t := l_t/|\mathcal {D}_t|$
Term length	$l_t := l_t/|\mathcal {T}_t|$ (noting that $|\mathcal {T}_t|=1$)

## Probabilistic derivation of IR models

To discuss the justification of ${\text {TF}}$ quantifications, we consider the probabilistic derivation of IR models. Most IR models can be derived from measuring the dependence between document and query. Let *d* denote a document, *q* a query, and *c* a collection. The document-query independence (DQI Roelleke and Wang 2008) is the point-wise mutual information expressed as:

24 $$\begin{aligned} {\text {DQI}}(d,q):= \log \left( \frac{P(d,q)}{P(d) \cdot P(q)}\right) \end{aligned}$$

Document and query are considered as sequences of term events. The decomposition of *d* leads to TF-IDF (and, for particular assumptions, to BM25), and the decomposition of *q* leads to LM. In this section we review the decomposition of *d*. When decomposing *d* using $P(d,q) = P(d|q)P(q)$ and then $P(d) = \prod _{t \in \mathcal {T}_d} P(t)^{\textit{tf}_d}$ and $P(d|q) = \prod _{t \in \mathcal {T}_d} P(t|q)^{\textit{tf}_d}$, we obtain:

25 $$\begin{aligned} \log \left( \frac{P(d|q)}{P(d)}\right) = \sum _{t \in \mathcal {T}_d} \textit{tf}_d\cdot \log \left( \frac{P(t|q)}{P(t)}\right) \end{aligned}$$

Here, *P*(*t*|*q*) is the query term probability, and *P*(*t*) is the background model (collection-wide) term probability. The equation makes two independence assumptions: different terms are independent, and also, the multiple occurrences of the same term are independent. The first assumption is reflected in applying the sum over different terms, and the second assumption is reflected by the total term frequency count, $\textit{tf}_d$.

To provide a justification for TF-IDF, one is looking for the bridges to close the gap between the probabilistic roots (assuming independence) and the TF-IDF. Expressed as an equation, we are looking for justifications to transform components of Eq. (25) to TF-IDF.

$$\begin{aligned} \begin{array}{ccc} \textit{tf}_d&{} \cdot &{} \log \frac{P(t|q)}{P(t)}\\ \downarrow &{} &{} \downarrow \\ {\text {TF}}(t,d) &{} \cdot &{} {\text {IDF}}(t)\\ \end{array} \end{aligned}$$

where ${\text {TF}}$ and ${\text {IDF}}$ are the two components, term frequency and inverse document frequency.

### Observations about the ${\text {TF}}$ component

The within-document term frequency ($\textit{tf}_d$) in IR models is usually not used pure due to its bias towards long documents as motivated in Sect. 2. The step from $\textit{tf}_d$ towards a quantification function involves a normalization component, referred to as $K_d$. The widely known $\text {TF}_{\text {BM25}}$ normalization factor is:

26 $$\begin{aligned} K_d = k_1 \cdot \left( 1-b + b \cdot \hat{l}_d \right) \end{aligned}$$

Given that $k_1$ and *b* are parameters of $K_d$, one should use the notation $K_{k_1,b,d}$, but for readability, we simplify the notation to $K_d$.

The following definition formalizes the well-defined spectrum of ${\text {TF}}$ quantifications (Roelleke et al. 2015).

#### **Definition 2**

($${\text {TF}}$$ Quantifications)

27 $$\begin{aligned} {\text {TF}}(t,d) = \left\{ \begin{array}{ll} \textit{tf}_d/K_d &{} \text {TF}_{\text {total}}\text {: independent}\\ \log (\textit{tf}_d/K_d+1) &{} \text {TF}_{\text {log}}\text {: logarithmic} \\ 2 \cdot \textit{tf}_d/(\textit{tf}_d+K_d) &{} \text {TF}_{\text {BM25}}\text {: semi-subsumed}\\ 1/K_d &{} \text {TF}_{\text {constant}}\text {: subsumed}\\ \end{array} \right. \end{aligned}$$

**Fig. 2:**
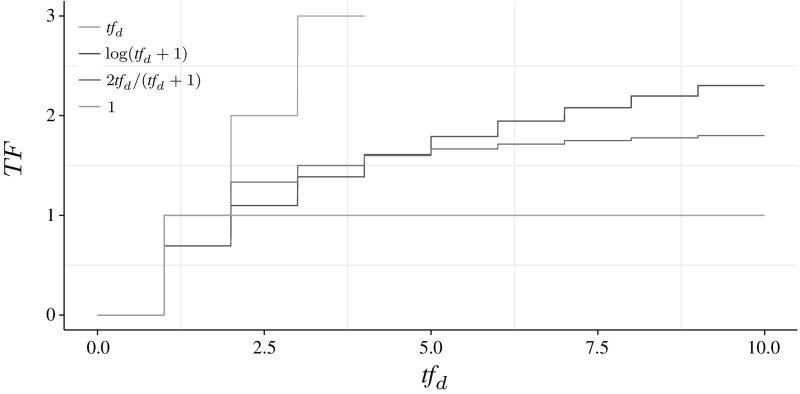
$${\text {TF}}$$ quantifications when $$K_d=1$$

The shape of the different ${\text {TF}}$ quantifications is shown in Fig. 2.

This spectrum is well-defined because each of these ${\text {TF}}$s correspond to an assumption regarding term events (Roelleke et al. 2015). $\text {TF}_{\text {total}}$ corresponds to assuming independence, and the $\text {TF}_{\text {log}}$ and $\text {TF}_{\text {BM25}}$ variants assume the occurrences of an event to be dependent.

With this understanding of what the TF stands for, namely a factor modeling a dependence assumption, the role of $K_d$ is to tune the dependence assumption. For $K_d>1$, that is for long documents, ${\text {TF}}(t,d)$ decreases, i.e. the dependence increases. This means that in long documents, the multiple term occurrences are more dependent than in short documents. This makes perfect sense when imagining a long document that repeats some terms many times.

This discussion makes evident that it is not just the length of the document that matters. To illustrate, consider two documents of equal length, for example, $l_d=300$ words. The standard $K_d$ will be equal for both documents. One document, however, contains many repetitions of some words (the document is verbose), whereas the other document contains many different words (the document is not verbose). Indeed, it is the verboseness and not simply the document length that leads to high term frequencies, and thus, to dependencies of multiple term occurrences. Therefore, this paper views $K_d$ as a combination of

the pivoted document length (${\text {pivdl}}$) and

the pivoted document verboseness ($\text {pivdv}$).

The following equation indicates the difference between the standard $K_d$ as known for BM25 [as shown in Eq. (26)], and the systematic extension proposed and investigated in this paper:

28 $$\begin{aligned} K_d = k_1 \cdot f({\text {pivdl}},\text {pivdv}) \end{aligned}$$

Here, $f({\text {pivdl}},\text {pivdv})$ is a function combining the two parameters, and this paper explores both a conjunctive and a disjunctive combination.

### Observations about the ${\text {IDF}}$ component

Regarding $\text {TF}_\text {BM25}\text {-IDF}$, the question remains of how to close the gap between *P*(*t*|*q*)/*P*(*t*) and ${\text {IDF}}$, as commonly defined in the literature: ${\text {IDF}}(t)=1/P_D(t)$. Mathematically, we are looking for a justification that leads to the following equation:

29 $$\begin{aligned} \log \left( \frac{P(t|q,c)}{P(t|c)}\right) = {\left\{ \begin{array}{ll} \log {\left( \frac{1}{P_D(t|c)}\right) } &{} t \in \mathcal {T}_q \\ 0 &{} t \not \in \mathcal {T}_q\\ \end{array}\right. } \end{aligned}$$

where in order to avoid confusion in the next derivation steps the collection symbol *c* is made explicit. We note that *P*(*t*|*c*) and $P_D(t|c)$ are both in the denominators of the functions. Let us consider what the relation between these two elements is, i.e. $P(t|c)/P_D(t|c)$. Referring back to the notations introduced at the end of Sect. 1.1, we have:

30 $$\begin{aligned} \frac{P_D(t|c)}{P(t|c)}= \frac{|\mathcal {D}_t|}{|\mathcal {D}|}\cdot \frac{l_c}{l_t}= \frac{l_c}{|\mathcal {D}|}\cdot \frac{|\mathcal {D}_t|}{l_t}= \frac{\bar{l}_d}{b_t} \end{aligned}$$

that is,

31 $$\begin{aligned} P_D(t|c)=\frac{\bar{l}_d}{b_t}\cdot P(t|c) \end{aligned}$$

and, substituting in the left side of (29), it becomes:

32 $$\begin{aligned} \log \left( \frac{P(t|q,c)}{P(t|c)}\right) = \log \left( \frac{P(t|q,c)}{b_t/\bar{l}_d\cdot P_D(t|c)}\right) \end{aligned}$$

This equation makes burstiness explicit, and in particular its otherwise implicit role in the relationship between IDF and the probabilistic model. If we were to return to Eq. (29), we are forced to consider:

33 $$\begin{aligned} P(t|q,c) = \left\{ \begin{array}{ll} b_t/\bar{l}_d &{} t \in \mathcal {T}_q\\ b_t/\bar{l}_d\cdot P_D(t|c) &{} t\not \in \mathcal {T}_q\\ \end{array} \right. = \left\{ \begin{array}{ll} b_t/\bar{l}_d &{} t \in \mathcal {T}_q\\ P(t|c) &{} t\not \in \mathcal {T}_q\\ \end{array} \right. \end{aligned}$$

Essentially, we have observed that the IDF, in its generic form of $1/P_D(t|c)$ implies that, when the term is not part of the query *q*, we estimate *P*(*t*|*q*) as the probability of the term in the collection (*P*(*t*|*c*)) and when the term is part of *q* we estimate it as $P(t|q)=b_t/\bar{l}_d$.

This separation between the cases when $t\in \mathcal {T}_q$ and $t\not \in \mathcal {T}_q$ is reminiscent of smoothing in language modeling. We could for instance write

34 $$\begin{aligned} P(t|q,c) = \lambda _q \, b_t/\bar{l}_d + (1 - \lambda _q) \, P(t|c) \end{aligned}$$

with

35 $$\begin{aligned} \lambda _q = \left\{ \begin{array}{ll} 1 &{} t \in \mathcal {T}_q\\ 0 &{} t\not \in \mathcal {T}_q\\ \end{array} \right. \end{aligned}$$

We shall call this an *extreme mixture*.

If we were to continue this inspiration from language modeling, leaving the above for a moment aside, to compute the *P*(*t*|*q*, *c*) we would estimate it through a linear mixture between the *P*(*t*|*c*) and the *P*(*t*|*q*), as follows:

36 $$\begin{aligned} P(t|q,c) = \lambda _q \, P(t|q) + (1 - \lambda _q) \, P(t|c) \end{aligned}$$

This equation is traditionally made because to estimate the probability of a term given the query *q*, when *q* is short, is not reliable (even more so than when considering a document *d*).

Substituting Eq. (36) into Eq. (32), we have:

37 $$\begin{aligned} \log {\left( \frac{P(t|q,c)}{b_t/\bar{l}_d\cdot P_D(t|c)}\right) } = \log {\left( (1-\lambda _q) + \lambda _q\frac{P(t|q)}{b_t/\bar{l}_d \cdot P_D(t|c)}\right) } \end{aligned}$$

where *P*(*t*|*q*) is calculated in a traditional way with a maximum likelihood estimator. However, this would not solve our problem given by the shortness of *q*. Instead, we need to use the estimation of Eq. (34). Then, reintroducing the distinction between $t\in \mathcal {T}_q$ and $t\not \in \mathcal {T}_q$ (i.e. $\lambda _q$), we obtain

38 $$\begin{aligned} \log {\left( (1-\lambda _q) + \lambda _q\frac{P(t|q)}{b_t/\bar{l}_d\cdot P_D(t|c)}\right) } = \left\{ \begin{array}{ll} \log {\left( (1-\lambda _q) + \lambda _q\frac{1}{P_D(t|c)}\right) } &{} t \in \mathcal {T}_q\\ 0 &{} t\not \in \mathcal {T}_q\\ \end{array} \right. \end{aligned}$$

In which if we set $\lambda _q=1$ then the foreground probability *P*(*t*|*c*) cancels out from the linear mixture assumption ending up with the standard ${\text {IDF}}$. We shall call this inverse document frequency $\text {IDF}_{\text {L}}$, where L stands for linear mixture, in contrast to the standard IDF (or $\text {IDF}_{\text {E}}$) that is defined by an extreme mixture.

### LM and TF-IDF

We already reached with our analysis a point where the border between LM and TF-IDF gets blurred. In this section we discuss the derivation of the LM model and highlight some commonality with the derivation of TF-IDF done in the previous section. We remember that the discussion of IDF in $\text {TF}_{\text {BM25}}$-IDF was started from Eq. (24), where we decomposed $P(d,q)=P(d|q)P(q)$. Here we can review the decomposition of *q* as $P(d,q) = P(q|d)P(d)$. We will then have: $P(q|d)=\prod _{t \in \mathcal {T}_q}P(t|d)^{\textit{tf}_q}$, and:

39 $$\begin{aligned} \log \left( \frac{P(q|d,c)}{P(q|c)}\right) = \sum _{t \in \mathcal {T}_q} \textit{tf}_q\cdot \log \left( \frac{P(t|d,c)}{P(t|c)}\right) \end{aligned}$$

Using again the observation formalized in Eq. (31), we observe the explicit presence of burstiness in the following equation, as it was in Eq. (32):

40 $$\begin{aligned} \log \left( \frac{P(t|d,c)}{P(t|c)}\right) = \log \left( \frac{P(t|d,c)}{b_t/\bar{l}_d \cdot P_D(t|c)}\right) \end{aligned}$$

Analogously for the derivation of TF-IDF for the estimation of *P*(*t*|*q*, *c*) in Eq. (36), and as commonly done in language modeling, we estimate the *P*(*t*|*d*, *c*) as:

$$\begin{aligned} P(t|d,c) = \lambda _{d} \, P(t|d) + (1-\lambda _{d}) \, P(t|c) \end{aligned}$$

and substituting to Eq. (40) we obtain:

41 $$\begin{aligned} \log \left( \frac{P(t|d,c)}{b_t/\bar{l}_d\cdot P_D(t|c)}\right) = \log \left( (1-\lambda _{d}) + \lambda _{d} \frac{P(t|d)}{b_t/\bar{l}_d\cdot P_D(t|c)} \right) \end{aligned}$$

We can now notice the symmetry with Eq. (37). In LM, when applying a Dirichlet-based mixture (D-LM), the value of $\lambda _{d}$ is Zhai and Lafferty (2001):

$$\begin{aligned} \lambda _{d} = \frac{l_d}{l_d+\mu } \end{aligned}$$

where $\mu$ is a parameter of the collection. This parameter could be set based on the average documents length $\bar{l}_d$. Zhai and Lafferty (2001) report values of $\mu \approx 2000$, though they note that the range of optimal parameter values in different collections is quite large (500–10,000). Later, Fang et al. (2004) posited that $\mu$ needs to be at least as large as the average document length ($\bar{l}_d$), so a reasonable value form for $\lambda _{d}$ is:

$$\begin{aligned} \lambda _{d} = \frac{l_d}{l_d+\bar{l}_d} = \frac{\frac{l_d}{\bar{l}_d}}{\frac{l_d}{\bar{l}_d} + 1} = \frac{{\text {pivdl}}}{{\text {pivdl}}+ 1} \end{aligned}$$

Now, just as we did for the normalization of ${\text {TF}}$ in the TF-IDF derivation, we should consider here not only the presence of the document length but also that of verboseness:

42 $$\begin{aligned} \lambda _{d} = \frac{f({\text {pivdl}},\text {pivdv})}{f({\text {pivdl}},\text {pivdv}) + 1} \end{aligned}$$

In a symmetric way we may define for TF-IDF a parameter not strongly dependent by the presence or absence of the term in *q* (as it was the case in the extreme mixture observed in the previous section) but rather using the Dirichlet based smoothing approach and the maximum likelihood estimation for $P(t|q) = \textit{tf}_q/l_q$:

43 $$\begin{aligned} \lambda _{q} = \frac{f({\text {pivql}},\text {pivqv})}{f({\text {pivql}},\text {pivqv}) + 1} \end{aligned}$$

However, the components of this formulation for $\lambda _{q}$ are generally not very informative (queries tend to be significantly shorter than documents, and therefore we cannot really talk about the verboseness of a query). Instead, at this place we can exploit the duality of document verboseness and length with term length and burstiness (see Sect. 3.3):

44 $$\begin{aligned} \lambda _{q} = \frac{f(\text {pivtl},\text {pivtb})}{f(\text {pivtl},\text {pivtb}) + 1} \end{aligned}$$

In summary, in this section we have explored the relationship between TF-IDF and LM. Both models apply a mixture: TF-IDF for estimating *P*(*t*|*q*, *c*), and LM for estimating *P*(*t*|*d*, *c*). Moreover, both models involve the component $b_t/\bar{l}_d \cdot P_D(t)$ measuring the discriminativeness of the term, where burstiness is made explicit.

The mixture assumption for *P*(*t*|*q*, *c*) leads to IDF and it becomes clear why IDF is seen as capturing burstiness in an “implicit” way (Church and Gale 1999). The Dirichlet-based mixture for *P*(*t*|*d*, *c*), usually only associated with the document length, is extended with the document verboseness. This extension is done analogously to the way the TF quantification has been extended for the TF-IDF models.

## Experiments

In this section, we first present the material, then the experimental setup. Finally we discuss the results.

**Table 2:** Test collection’s information about the collection size $|\mathcal {D}|$, number of terms $|\mathcal {T}|$, collection length $l_c$, average document length $\bar{l}_d$, average verboseness $\bar{v}_d$, elite average verboseness $\breve{v}_d$, average term length $\bar{l}_t$, average burstiness $\bar{b}_t$, and elite average burstiness $\breve{b}_t$

Corpus	EC	Challenge	$|\mathcal {D}|$	$|\mathcal {T}|$	$l_c$
			$\bar{l}_d$	$\bar{v}_d$	$\breve{v}_d\downarrow$
			$\bar{l}_t$	$\bar{b}_t$	$\breve{b}_t$
Aquaint	TREC	HARD’05	1,033,461	647,280	282,858,247
			273.700	436.995	1.519
			436.995	273.700	1.384
Disks 4&5	TREC	Ad Hoc 8	528,106	737,963	156,226,039
			295.823	211.699	1.575
			211.699	295.823	1.377
eHealth’14	CLEF	eHealth’14	1,104,298	1,103,947	685,458,908
			620.917	308.294	1.900
			308.294	620.917	1.349
.GOV	TREC	Web’02	1,214,592	2,937,251	1,770,120,644
			1,457.379	602.645	4.830
			602.645	1,457.379	3.012

Ordered as indicated by the arrow ($\downarrow$)

### Setup and materials

To test the ${\text {TF}}$ normalization variants on the different kinds of ${\text {TF}}$ quantifications, we used 4 test collections: TREC HARD 2005, TREC Ad Hoc 8, CLEF eHealth 2014, and TREC Web 2002. Details and corpora properties shown in Table 2. The test collections have been purposefully chosen with a high degree of variability of $\breve{v}_d$. In this way we can observe the different use of the language in different domains (e.g. we observe that in .GOV on average a term is repeated 218% more times than in the Aquaint collection). We developed^2^ the tested IR models on the IR platform Terrier^3^ 4.2. All the documents have been preprocessed using the English tokenizer and Porter stemmer of the Terrier search engine. All the topics, when multiple lengths are available in the test collections, are of the shortest kind.

We tested a total of 24 models:

- 16 models based on TF-IDF variants: 4 ${\text {TF}}$ normalizations for each of the 4 ${\text {TF}}$ quantifications defined in Definition 2. Each model is identified by its ${\text {TF}}$ quantification, $\text {TF}_{\text {total}}$, $\text {TF}_{\text {log}}$, $\text {TF}_{\text {BM25}}$, and $\text {TF}_{\text {constant}}$ and kind of ${\text {TF}}$ normalization applied: non-elite disjunctive $\ddot{K}_{\vee ,d}$, non-elite conjunctive $\ddot{K}_{\wedge ,d}$, elite disjunctive $\hat{K}_{\vee ,d}$ and elite conjunctive $\hat{K}_{\wedge ,d}$.
- 4 models based on D-LM: Each Dirichlet-based mixture is identified by its kind of $\lambda _{d}$ normalization applied: non-elite disjunctive $\ddot{\lambda }_{\vee ,d}$, non-elite conjunctive $\ddot{\lambda }_{\wedge ,d}$, elite disjunctive $\hat{\lambda }_{\vee ,d}$ and elite conjunctive $\hat{\lambda }_{\wedge ,d}$.
- 4 models based on the TF-$\text {IDF}_\text {L}$: Each Dirichlet-based mixture is identified by its kind of $\lambda _{q}$ normalization applied: non-elite disjunctive $\ddot{\lambda }_{\vee ,q}$, non-elite conjunctive $\ddot{\lambda }_{\wedge ,q}$, elite disjunctive $\hat{\lambda }_{\vee ,q}$ and elite conjunctive $\hat{\lambda }_{\wedge ,q}$. As ${\text {TF}}$ component, we select the non-normalized $\text {TF}_{\text {total}}$.

The TF normalization of each model presents 3 parameters: $k_1$, *b* and the new *a* introduced in this paper. The D-LM and TF-$\text {IDF}_\text {L}$ based models present 2 parameters: *b* and *a*. Our experiments focus on the parameter *a*. For $k_1$ and *b*, there are two ways of selecting their values: using the standard values from the literature, or identifying trained values. For the models based on the TF-IDF variants, the standard parameters for $\text {TF}_{\text {BM25}}$ are $k_1=1.2$ and $b=0.7$ (Robertson et al. 1994). The standard parameter for $\text {TF}_{\text {total}}$ and $\text {TF}_{\text {constant}}$ is $b=0$ that simplifies $K_d$ to a constant. In this case we set $k_1=1$, because it is easy to demonstrate that to change the parameter $k_1$, as long as $k_1>0$, does not change the rank of the retrieved documents for these two quantifications. The same set of parameter values are set for the standard $\text {TF}_{\text {log}}$ ($b=0$, $k_1=1$). For the models based on the D-LM, the standard parameters are $k_1=1$ and $b=0$, which reduces to the standard definition of D-LM (Zhai and Lafferty 2001). For the models based on the LM variant derived by TF-IDF, the standard parameters are $k_1=+\infty$, which reduces to the standard TF-IDF model with non normalized $\text {TF}_{\text {total}}$ quantification.

To identify trained values, the parameters of each model have been spanned as follows: $a,b \in [0, 1]$ at steps of 0.1, and $k_1 \in [0,5]$, from 0 to 1 at steps decided by the function 1 / *n* with $n \in \{1,...,50\}$, and from 1 to 5 at steps of 0.1. The trained values are obtained maximizing the mean over the topics of the selected evaluation measure. For every model’s configuration that requires training we perform a fivefold cross validation.

The IR evaluation measures employed are $\text {AP}$, $\text {NDCG}$ and $\text {P@10}$.

### Model candidates/structure

Each TF-IDF model candidate is characterized by choosing one of the following options:

1. Pivotization: elite pivotization or non-elite pivotization for document verboseness and length;
2. Normalization: conjunctive ($\wedge$) or disjunctive ($\vee$) combination of pivoted document verboseness and length into $K_d$;
3. Quantification: $$\text {TF}_{\text {total}}$$, $$\text {TF}_{\text {log}}$$, $$\text {TF}_{\text {BM25}}$$, or $$\text {TF}_{\text {constant}}$$;
4. Parameter Settings: standard (S) or trained (T) parameters.

Each D-LM model candidate is characterized by choosing one of the following options:

1. Pivotization: elite pivotization or non-elite pivotization for document verboseness and length;
2. Normalization: conjunctive ($\wedge$) or disjunctive ($\vee$) combination of pivoted document verboseness and length into $\lambda _d$;
3. Parameter Settings: standard (S) or trained (T) parameters.

Each TF-$\text {IDF}_{\text {L}}$ model candidate is characterized by choosing one of the following options:

1. Pivotization: elite pivotization or non-elite pivotization for term length and burstiness;
2. Normalization: conjunctive ($\wedge$) or disjunctive ($\vee$) combination of pivoted term length and burstiness into $\lambda _q$;
3. Parameter Settings: standard (S) or trained (T) parameters.

### Results

**Fig. 3:**
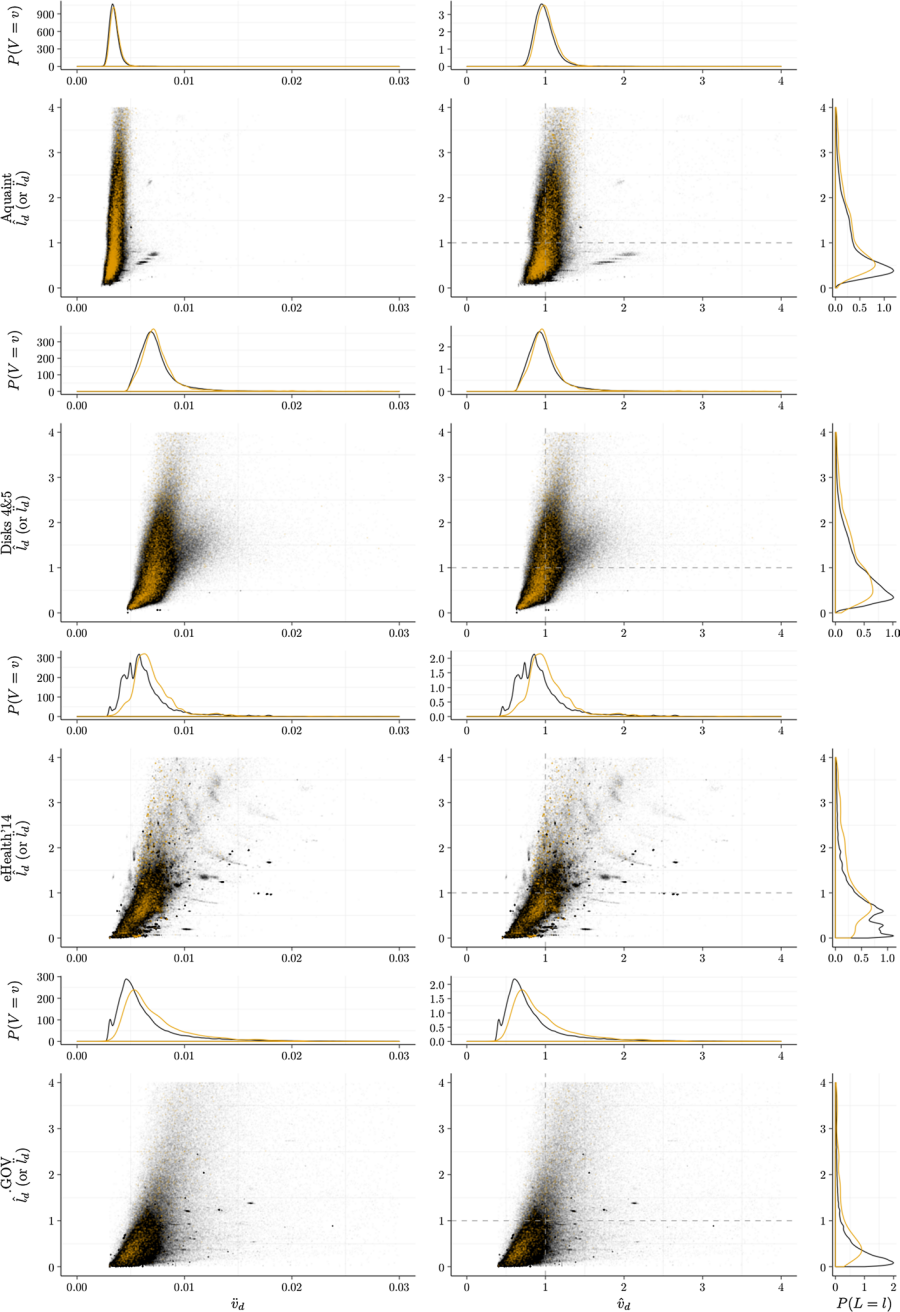
Distribution of verboseness in the x-axis and document length in the y-axis of the relevant documents (in gold) and all the documents (in black). Left plot shows the non-elite pivotization case of verboseness ($\ddot{v}_d$) and length ($\ddot{l}_d$) and the right plot shows the elite pivotization case of verboseness ($\hat{v}_d$) and length ($\hat{l}_d$)

The main results observed are:

1. Document Verboseness versus Length: show a certain independence as shown by the shape of the distributions in Fig. 3;
2. Pivotization: for TF-IDF models the elite pivotization is overall better than the non-elite one; for D-LM models the non-elite pivotization performs better.
3. Normalization: for TF-IDF models the combination of document verboseness and length achieves significantly better results, especially when combined in a conjunctive fashion; for D-LM models the combination of document verboseness and length rarely achieves statistically significance;
4. TF-Quantification: $\text {TF}_{\text {BM25}}$ appears best, with $\text {TF}_{\text {log}}$ close behind;
5. Standard versus Trained parameter: in both parameter configurations, standard and trained, the use of verboseness makes the model achieve better results. On the other hand, the use of term length most of the time has a negligible impact.

**Table 3:** Comparison of the scores obtained with the TF-IDF model candidates with each ${\text {TF}}$ normalization using the non-elite and elite pivotization for the HARD 2005 test collection

P	Q	K	C	k1	b	a	$\text {AP}$	$\text {NDCG}$	$\text {P@10}$
Non-elite	$\text {TF}_{\text {total}}$	S	–	$>0$	0.0	–	0.0721	0.2936	0.1920
		T	–	$>0$	0.5	–	0.0900 $\dagger$	0.3201 $\dagger$	0.2160
			$\vee$	$>0$	0.9	0.9	0.0904 $\dagger$	0.3223 $\dagger \, \ddagger$	0.2200
			$\wedge$	$>0$	1.0	0.6	0.0942 $\dagger \, \ddagger$	0.3277 $\dagger \, \ddagger$	0.2380 $\ddagger$
	$\text {TF}_{\text {log}}$	S	–	1.0	0.0	–	0.1614	0.4424	0.4160
		T	–	0.2	0.3	–	0.2005 $\dagger$	0.4799 $\dagger$	0.4360
			$\vee$	0.2	0.4	0.2	0.2010 $\dagger$	0.4801 $\dagger$	0.4320
			$\wedge$	5.0	0.8	0.7	0.2003 $\dagger$	0.4813 $\dagger$	0.4400
	$\text {TF}_{\text {BM25}}$	S	–	1.2	0.7	–	0.1848	0.4563	0.3660
		T	$\vee$	1.2	0.7	0.6	0.1898	0.4584	0.4280 $\dagger$
			–	1.5	0.3	–	0.2023 $\dagger$	0.4797 $\dagger$	0.4440 $\dagger$
			$\vee$	1.9	0.4	0.5	0.2030 $\dagger$	0.4802 $\dagger$	0.4480 $\dagger$
			$\wedge$	3.2	0.4	0.3	**0.2032** $\dagger$	**0.4812** $\dagger$	**0.4540** $\dagger$
	$\text {TF}_{\text {constant}}$	S	–	$>0$	0.0	–	0.0613	0.2436	0.1500
		T	–	$>0$	0.1	–	0.0735 $\dagger$	0.2744 $\dagger$	0.1620
			$\vee$	$>0$	0.2	0.7	0.0742 $\dagger$	0.2756 $\dagger$	0.1620
			$\wedge$	$>0$	0.1	0.0	0.0740 $\dagger$	0.2745 $\dagger$	0.1660
Elite	$\text {TF}_{\text {total}}$	S	–	$>0$	0.0	–	0.0721	0.2936	0.1920
		T	–	$>0$	0.5	–	0.0900 $\dagger$	0.3201 $\dagger$	0.2160
			$\vee$	$>0$	1.0	0.6	0.0946 $\dagger \, \ddagger$	0.3283 $\dagger \, \ddagger$	0.2380 $\ddagger$
			$\wedge$	$>0$	1.0	0.6	0.0942 $\dagger \, \ddagger$	0.3277 $\dagger \, \ddagger$	0.2380 $\ddagger$
	$\text {TF}_{\text {log}}$	S	–	1.0	0.0	–	0.1614	0.4424	0.4160
		T	–	0.2	0.3	–	0.2005 $\dagger$	0.4799 $\dagger$	0.4360
			$\vee$	0.2	0.6	0.5	0.2013 $\dagger$	0.4798 $\dagger$	0.4300
			$\wedge$	0.2	0.8	0.7	0.2003 $\dagger$	0.4810 $\dagger$	0.4400
	$\text {TF}_{\text {BM25}}$	S	–	1.2	0.7	–	0.1848	0.4563	0.3660
		T	$\vee$	1.2	0.7	0.6	0.2012 $\dagger$	0.4759 $\dagger$	**0.4480** $\dagger$
			–	1.5	0.3	–	0.2023 $\dagger$	0.4797 $\dagger$	0.4440 $\dagger$
			$\vee$	1.5	0.5	0.5	0.2034 $\dagger$	0.4807 $\dagger$	0.4420 $\dagger$
			$\wedge$	1.9	0.8	0.7	**0.2037** $\dagger$	**0.4833** $\dagger$	0.4400 $\dagger$
	$\text {TF}_{\text {constant}}$	S	–	$>0$	0.0	–	0.0613	0.2436	0.1500
		T	–	$>0$	0.1	–	0.0735 $\dagger$	0.2744 $\dagger$	0.1620
			$\vee$	$>0$	0.1	0.0	0.0735 $\dagger$	0.2744 $\dagger$	0.1620
			$\wedge$	$>0$	0.1	0.0	0.0740 $\dagger$	0.2745 $\dagger$	0.1660

Column K indicates if standard (S) or trained (T) parameters are used. $\dagger$ indicates statistical significance (paired t-test, $p<0.05$) against the standard and $\ddagger$ against the trained parameters when *a* is not used

**Table 4:** Comparison of the scores obtained with the TF-IDF model candidates with each ${\text {TF}}$ normalization using the non-elite and elite pivotization for the Ad Hoc 8 test collection

P	Q	K	C	k1	b	a	$\text {AP}$	$\text {NDCG}$	$\text {P@10}$
Non-elite	$\text {TF}_{\text {total}}$	S	–	$>0$	0.0	–	0.0635	0.2762	0.1360
		T	–	$>0$	0.5	–	0.0977 $\dagger$	0.3306 $\dagger$	0.2240 $\dagger$
			$\vee$	$>0$	0.5	0.0	0.0977 $\dagger$	0.3306 $\dagger$	0.2240 $\dagger$
			$\wedge$	$>0$	1.0	0.5	0.1076 $\dagger \, \ddagger$	0.3491 $\dagger \, \ddagger$	0.2400 $\dagger$
	$\text {TF}_{\text {log}}$	S	–	1.0	0.0	–	0.1753	0.4568	0.3360
		T	–	0.1	0.3	–	0.2478 $\dagger$	0.5381 $\dagger$	0.4280 $\dagger$
			$\vee$	0.1	0.9	0.9	0.2563	0.5415	0.4560
			$\wedge$	0.1	0.9	0.5	0.2625 $\dagger \, \ddagger$	0.5475 $\dagger$	0.4620 $\dagger \, \ddagger$
	$\text {TF}_{\text {BM25}}$	S	–	1.2	0.7	–	0.2433	0.5193	0.4680
		T	$\vee$	1.2	0.7	0.8	0.2614 $\dagger$	0.5438 $\dagger$	0.4480
			–	0.6	0.3	–	0.2614 $\dagger$	0.5447 $\dagger$	0.4520
			$\vee$	0.6	0.3	0.1	0.2616 $\dagger$	0.5441 $\dagger$	0.4620 $\ddagger$
			$\wedge$	2.7	0.6	0.5	**0.2681** $\dagger \, \ddagger$	**0.5523** $\dagger \, \ddagger$	**0.4660**
	$\text {TF}_{\text {constant}}$	S	–	$>0$	0.0	–	0.1550	0.4071	0.2060
		T	–	$>0$	0.1	–	0.1868 $\dagger$	0.4387 $\dagger$	0.3260 $\dagger$
			$\vee$	$>0$	0.1	0.9	0.1880 $\dagger$	0.4452 $\dagger \, \ddagger$	0.3240 $\dagger$
			$\wedge$	$>0$	0.2	0.4	0.1922 $\dagger$	0.4462 $\dagger \, \ddagger$	0.3260 $\dagger$
Elite	$\text {TF}_{\text {total}}$	S	–	$>0$	0.0	–	0.0635	0.2762	0.1360
		T	–	$>0$	0.5	–	0.0977 $\dagger$	0.3306 $\dagger$	0.2240 $\dagger$
			$\vee$	$>0$	1.0	0.7	0.1056 $\dagger \, \ddagger$	0.3469 $\dagger \, \ddagger$	0.2380 $\dagger$
			$\wedge$	$>0$	1.0	0.5	0.1076 $\dagger \, \ddagger$	0.3491 $\dagger \, \ddagger$	0.2400 $\dagger$
	$\text {TF}_{\text {log}}$	S	–	1.0	0.0	–	0.1753	0.4568	0.3360
		T	–	0.1	0.3	–	0.2478 $\dagger$	0.5381 $\dagger$	0.4280 $\dagger$
			$\vee$	0.1	1.0	0.7	0.2521 $\dagger$	0.5435 $\dagger$	0.4500 $\dagger \, \ddagger$
			$\wedge$	0.1	0.8	0.6	0.2562 $\dagger \, \ddagger$	0.5474 $\dagger \, \ddagger$	0.4540 $\dagger \, \ddagger$
	$\text {TF}_{\text {BM25}}$	S	–	1.2	0.7	–	0.2433	0.5193	0.4680
		T	$\vee$	1.2	0.7	0.6	0.2535 $\dagger$	0.5399 $\dagger$	**0.4700**
			–	0.6	0.3	–	0.2614 $\dagger$	0.5447 $\dagger$	0.4520
			$\vee$	0.5	1.0	0.7	0.2638 $\dagger$	0.5463 $\dagger$	**0.4700**
			$\wedge$	0.6	0.6	0.5	**0.2681** $\dagger \, \ddagger$	**0.5524** $\dagger \, \ddagger$	0.4680 $\ddagger$
	$\text {TF}_{\text {constant}}$	S	–	$>0$	0.0	–	0.1550	0.4071	0.2060
		T	–	$>0$	0.1	–	0.1868 $\dagger$	0.4387 $\dagger$	0.3260 $\dagger$
			$\vee$	$>0$	0.1	0.4	0.1878 $\dagger$	0.4418 $\dagger \, \ddagger$	0.3320 $\dagger$
			$\wedge$	$>0$	0.2	0.4	0.1922 $\dagger$	0.4462 $\dagger \, \ddagger$	0.3260 $\dagger$

Column K indicates if standard (S) or trained (T) parameters are used. $\dagger$ indicates statistical significance (paired t-test, $p<0.05$) against the standard and $\ddagger$ against the trained parameters when *a* is not used

**Table 5:** Comparison of the scores obtained with the TF-IDF model candidates with each ${\text {TF}}$ normalization using the non-elite and elite pivotization for the eHealth 2014 test collection

P	Q	K	C	k1	b	a	$\text {AP}$	$\text {NDCG}$	$\text {P@10}$
Non-elite	$\text {TF}_{\text {total}}$	S	–	$>0$	0.0	–	0.1166	0.3361	0.2640
		T	–	$>0$	0.7	–	0.2594 $\dagger$	0.5206 $\dagger$	0.5580 $\dagger$
			$\vee$	$>0$	0.8	0.4	0.2610 $\dagger$	0.5209 $\dagger$	0.5540 $\dagger$
			$\wedge$	$>0$	1.0	0.4	0.2699 $\dagger$	0.5322 $\dagger$	0.5580 $\dagger$
	$\text {TF}_{\text {log}}$	S	–	1.0	0.0	–	0.2106	0.4637	0.4280
		T	–	0.2	0.7	–	0.4222	0.6701 $\dagger$	0.7960 $\dagger$
			$\vee$	0.4	0.8	0.5	0.4242	**0.6729** $\dagger \, \ddagger$	0.8000 $\dagger$
			$\wedge$	1.9	1.0	0.4	**0.4260**	**0.6729** $\dagger$	**0.8040** $\dagger$
	$\text {TF}_{\text {BM25}}$	S	–	1.2	0.7	–	0.3729	0.6310	0.7640
		T	$\vee$	1.2	0.7	0.0	0.3729	0.6310	0.7640
			–	4.5	0.6	–	0.4022 $\dagger$	0.6595 $\dagger$	0.7840
			$\vee$	4.5	0.6	0.0	0.4022 $\dagger$	0.6595 $\dagger$	0.7840
			$\wedge$	4.5	0.7	0.0	0.4018 $\dagger$	0.6542 $\dagger$	0.7880
	$\text {TF}_{\text {constant}}$	S	–	$>0$	0.0	–	0.0474	0.2021	0.1140
		T	–	$>0$	0.2	–	0.0755 $\dagger$	0.2552 $\dagger$	0.2280 $\dagger$
			$\vee$	$>0$	0.0	0.0	0.0840 $\dagger$	0.3523 $\dagger \, \ddagger$	0.1760 $\dagger$
			$\wedge$	$>0$	0.2	0.2	0.0745 $\dagger$	0.2551 $\dagger$	0.2260 $\dagger$
Elite	$\text {TF}_{\text {total}}$	S	–	$>0$	0.0	–	0.1166	0.3361	0.2640
		T	–	$>0$	0.7	–	0.2594 $\dagger$	0.5206 $\dagger$	0.5580 $\dagger$
			$\vee$	$>0$	1.0	0.5	0.2697 $\dagger$	0.5316 $\dagger \, \ddagger$	0.5820 $\dagger$
			$\wedge$	$>0$	1.0	0.4	0.2699 $\dagger$	0.5322 $\dagger$	0.5580 $\dagger$
	$\text {TF}_{\text {log}}$	S	–	1.0	0.0	–	0.2106	0.4637	0.4280
		T	–	0.2	0.7	–	0.4222	0.6701 $\dagger$	0.7960 $\dagger$
			$\vee$	0.2	1.0	0.4	**0.4239**	0.6713 $\dagger$	**0.8080** $\dagger$
			$\wedge$	0.2	1.0	0.4	**0.4239**	**0.6715** $\dagger$	0.8060 $\dagger$
	$\text {TF}_{\text {BM25}}$	S	–	1.2	0.7	–	0.3729	0.6310	0.7640
		T	$\vee$	1.2	0.7	0.1	0.3742	0.6320	0.7640
			–	4.5	0.6	–	0.4022 $\dagger$	0.6595 $\dagger$	0.7840
			$\vee$	5.0	1.0	0.5	0.4079 $\dagger \, \ddagger$	0.6635 $\dagger \, \ddagger$	0.7900
			$\wedge$	5.0	1.0	0.4	0.4092 $\dagger \, \ddagger$	0.6607 $\dagger$	0.8000
	$\text {TF}_{\text {constant}}$	S	–	$>0$	0.0	–	0.0474	0.2021	0.1140
		T	–	$>0$	0.2	–	0.0755 $\dagger$	0.2552 $\dagger$	0.2280 $\dagger$
			$\vee$	$>0$	0.2	0.0	0.0755 $\dagger$	0.2552 $\dagger$	0.2280 $\dagger$
			$\wedge$	$>0$	0.2	0.2	0.0745 $\dagger$	0.2551 $\dagger$	0.2260 $\dagger$

Column K indicates if standard (S) or trained (T) parameters are used. $\dagger$ indicates statistical significance (paired t-test, $p<0.05$) against the standard and $\ddagger$ against the trained parameters when *a* is not used

**Table 6:** Comparison of the scores obtained with the TF-IDF model candidates with each ${\text {TF}}$ normalization using the non-elite and elite pivotization for the Web 2002 test collection

P	Q	K	C	k1	b	a	$\text {AP}$	$\text {NDCG}$	$\text {P@10}$
Non-elite	$\text {TF}_{\text {total}}$	S	–	$>0$	0.0	–	0.0171	0.1387	0.0260
		T	–	$>0$	0.9	–	0.0568 $\dagger$	0.2642 $\dagger$	0.0880 $\dagger$
			$\vee$	$>0$	0.9	0.4	0.0577 $\dagger$	0.2713 $\dagger \, \ddagger$	0.0820 $\dagger$
			$\wedge$	$>0$	1.0	0.4	0.0563 $\dagger$	0.2732 $\dagger$	0.0800 $\dagger$
	$\text {TF}_{\text {log}}$	S	–	1.0	0.0	–	0.0603	0.2719	0.1100
		T	–	0.2	0.8	-	0.1951 $\dagger$	0.4799 $\dagger$	0.2420 $\dagger$
			$\vee$	0.2	0.9	0.6	0.1991 $\dagger$	0.4803 $\dagger$	0.2360 $\dagger$
			$\wedge$	0.2	0.9	0.2	0.1974 $\dagger$	0.4812 $\dagger$	0.2360 $\dagger$
	$\text {TF}_{\text {BM25}}$	S	–	1.2	0.7	–	0.1948	0.4696	0.2380
		T	$\vee$	1.2	0.7	0.0	0.1948	0.4696	0.2380
			–	4.1	0.7	–	0.2010	0.4777	**0.2520**
			$\vee$	3.1	0.7	0.1	**0.2016**	**0.4816**	0.2420
			$\wedge$	5.0	0.8	0.2	0.1923	0.4722	0.2520
	$\text {TF}_{\text {constant}}$	S	–	$>0$	0.0	–	0.0140	0.1514	0.0140
		T	–	$>0$	0.1	–	0.0310 $\dagger$	0.2041 $\dagger$	0.0500 $\dagger$
			$\vee$	$>0$	0.2	0.3	0.0310 $\dagger$	0.2008 $\dagger$	0.0500 $\dagger$
			$\wedge$	$>0$	0.1	0.5	0.0311 $\dagger$	0.1979 $\dagger$	0.0480 $\dagger$
Elite	$\text {TF}_{\text {total}}$	S	–	$>0$	0.0	–	0.0171	0.1387	0.0260
		T	–	$>0$	0.9	–	0.0568 $\dagger$	0.2642 $\dagger$	0.0880 $\dagger$
			$\vee$	$>0$	1.0	0.4	0.0635 $\dagger$	0.2860 $\dagger \, \ddagger$	0.0940 $\dagger$
			$\wedge$	$>0$	1.0	0.4	0.0563 $\dagger$	0.2732 $\dagger$	0.0800 $\dagger$
	$\text {TF}_{\text {log}}$	S	–	1.0	0.0	–	0.0603	0.2719	0.1100
		T	–	0.2	0.8	-	0.1951 $\dagger$	0.4799 $\dagger$	0.2420 $\dagger$
			$\vee$	0.1	0.9	0.2	0.1989	**0.4817**	0.2360
			$\wedge$	0.1	0.9	0.2	0.1975 $\dagger$	0.4816 $\dagger$	0.2380 $\dagger$
	$\text {TF}_{\text {BM25}}$	S	–	1.2	0.7	–	0.1948	0.4696	0.2380
		T	$\vee$	1.2	0.7	0.0	0.1948	0.4696	0.2380
			–	4.1	0.7	–	0.2010	0.4777	**0.2520**
			$\vee$	3.6	0.8	0.2	**0.2016**	0.4808	0.2460
			$\wedge$	3.3	1.0	0.4	0.1966	0.4770	0.2500
	$\text {TF}_{\text {constant}}$	S	–	$>0$	0.0	–	0.0140	0.1514	0.0140
		T	–	$>0$	0.1	–	0.0310 $\dagger$	0.2041 $\dagger$	0.0500 $\dagger$
			$\vee$	$>0$	0.2	0.3	0.0319 $\dagger$	0.1988 $\dagger$	0.0520 $\dagger$
			$\wedge$	$>0$	0.1	0.5	0.0311 $\dagger$	0.1979 $\dagger$	0.0480 $\dagger$

Column K indicates if standard (S) or trained (T) parameters are used. $\dagger$ indicates statistical significance (paired t-test, $p<0.05$) against the standard and $\ddagger$ against the trained parameters when *a* is not used

**Table 7:** Comparison of the scores obtained with the D-LM models candidates using the non-elite and elite pivotization

Challenge	P	K	C	b	a	$\text {AP}$	$\text {NDCG}$	$\text {P@10}$
HARD’05		S	–	1.0	–	0.1912	0.4680	0.4220
	Non-elite	T	$\vee$	1.0	0.8	0.1970	0.4801 $\dagger$	**0.4580** $\dagger$
			$\wedge$	1.0	0.3	**0.1998** $\dagger$	**0.4806** $\dagger$	0.4380
	Elite	T	$\vee$	1.0	0.0	0.1912	0.4680	0.4220
			$\wedge$	1.0	0.0	0.1912	0.4680	0.4220
Ad Hoc 8		S	–	1.0	–	0.2583	0.5420	0.4560
	Non-elite	T	$\vee$	0.9	0.7	**0.2625** $\dagger$	**0.5481** $\dagger$	0.4600
			$\wedge$	0.8	0.3	0.2606	0.5448	0.4480
	Elite	T	$\vee$	0.9	0.0	0.2589	0.5410	**0.4680**
			$\wedge$	0.9	0.0	0.2587	0.5415	0.4600
eHealth’14		S	–	1.0	–	0.3863	0.6444	0.7980
	Non-elite	T	$\vee$	0.8	0.5	0.3965 $\dagger$	0.6468	0.7900
			$\wedge$	0.7	0.7	**0.4082** $\dagger$	**0.6616** $\dagger$	**0.7920**
	Elite	T	$\vee$	0.8	0.0	0.3939 $\dagger$	0.6467	0.7820 $\dagger$
			$\wedge$	0.7	0.0	0.3927 $\dagger$	0.6468	0.7900
Web’02		S	–	1.0	–	0.1877	0.4617	0.2380
	Non-elite	T	$\vee$	0.8	0.0	0.1984 $\dagger$	0.4767 $\dagger$	0.2580
			$\wedge$	0.5	0.1	**0.2039** $\dagger$	**0.4844** $\dagger$	0.2600
	Elite	T	$\vee$	0.9	0.3	0.2002 $\dagger$	0.4785 $\dagger$	0.2620
			$\wedge$	0.5	0.0	0.2037 $\dagger$	0.4836 $\dagger$	**0.2660**

Column K indicates if standard (S) or trained (T) parameters are used. $\dagger$ indicates statistical significance (t-test, $p<0.05$) against the standard parameters

**Table 8:** Comparison of the scores obtained with the TF-$\text {IDF}_\text {L}$ model candidates using the non-elite and elite pivotization

Challenge	P	K	C	b	a	$\text {AP}$	$\text {NDCG}$	$\text {P@10}$
HARD’05		S	–	–	–	0.0721	0.2936	0.1920
	Non-elite	T	$\vee$	1.0	1.0	**0.0967** $\dagger$	**0.3329** $\dagger$	**0.2120**
			$\wedge$	1.0	1.0	**0.0967** $\dagger$	**0.3329** $\dagger$	**0.2120**
	Elite	T	$\vee$	1.0	1.0	0.0753 $\dagger$	0.2994 $\dagger$	0.1960
			$\wedge$	1.0	1.0	0.0753 $\dagger$	0.2994 $\dagger$	0.1960
Ad Hoc 8		S	–	–	–	0.0635	0.2762	0.1360
	Non-elite	T	$\vee$	1.0	1.0	**0.1500** $\dagger$	**0.4135** $\dagger$	**0.2440** $\dagger$
			$\wedge$	1.0	1.0	**0.1500** $\dagger$	**0.4135** $\dagger$	**0.2440** $\dagger$
	Elite	T	$\vee$	1.0	1.0	0.0688 $\dagger$	0.2914 $\dagger$	0.1480 $\dagger$
			$\wedge$	1.0	1.0	0.0688 $\dagger$	0.2914 $\dagger$	0.1480 $\dagger$
eHealth’14		S	–	–	–	0.1166	0.3361	0.2640
	Non-elite	T	$\vee$	1.0	1.0	**0.1623** $\dagger$	**0.4177** $\dagger$	**0.3220**
			$\wedge$	1.0	1.0	**0.1623** $\dagger$	**0.4177** $\dagger$	**0.3220**
	Elite	T	$\vee$	1.0	1.0	0.1231 $\dagger$	0.3502 $\dagger$	0.2780
			$\wedge$	1.0	1.0	0.1231 $\dagger$	0.3502 $\dagger$	0.2780
Web’02		S	–	–	–	0.0171	0.1387	0.0260
	Non-elite	T	$\vee$	1.0	1.0	**0.0249** $\dagger$	**0.1865** $\dagger$	**0.0460** $\dagger$
			$\wedge$	1.0	1.0	**0.0249** $\dagger$	**0.1865** $\dagger$	**0.0460** $\dagger$
	Elite	T	$\vee$	1.0	1.0	0.0183 $\dagger$	0.1456 $\dagger$	0.0280
			$\wedge$	1.0	1.0	0.0183 $\dagger$	0.1456 $\dagger$	0.0280

Column K indicates if standard (S) or trained (T) parameters are used. $\dagger$ indicates statistical significance (t-test, $p<0.05$) against the standard

**Table 9:** Fivefold cross validation of the trained TF-IDF models candidates observed in Tables 3, 4, 5, and 6 for the evaluation measure $\text {AP}$

P	Q	C	k1	b	a	HARD’05	Ad Hoc 8	eHealth’14	Web’02
Non-elite	$\text {TF}_{\text {total}}$	–	$>0$	$*$	–	0.0873	0.0927	0.2594	0.0543
		$\vee$	$>0$	$*$	$*$	0.0873	0.0927	0.2594	0.0543
		$\wedge$	$>0$	$*$	$*$	0.0942	0.1058	0.2699	0.0523
	$\text {TF}_{\text {log}}$	–	$*$	$*$	–	0.2005	0.2436	0.4136	0.1911
		$\vee$	$*$	$*$	$*$	0.2293	0.2591	**0.6081**	**0.2058**
		$\wedge$	$*$	$*$	$*$	0.2257	0.2679	0.5985	0.2048
	$\text {TF}_{\text {BM25}}$	$\vee$	1.2	0.7	$*$	0.2228	**0.2718**	0.5679	0.2033
		–	$*$	$*$	–	0.1983	0.2597	0.3987	0.1937
		$\vee$	$*$	$*$	$*$	**0.2316**	0.2671	0.6050	0.2042
		$\wedge$	$*$	$*$	$*$	0.2006	0.2634	0.3990	0.1892
	$\text {TF}_{\text {constant}}$	–	$>0$	$*$	–	0.0735	0.1868	0.0727	0.0309
		$\vee$	$>0$	$*$	$*$	0.1215	0.2087	0.2647	0.0559
		$\wedge$	$>0$	$*$	$*$	0.0740	0.1881	0.0735	0.0291
Elite	$\text {TF}_{\text {total}}$	–	$>0$	$*$	–	0.0873	0.0927	0.2594	0.0543
		$\vee$	$>0$	$*$	$*$	0.1495	0.1206	0.5188	0.0965
		$\wedge$	$>0$	$*$	$*$	0.0942	0.1058	0.2699	0.0523
	$\text {TF}_{\text {log}}$	–	$*$	$*$	–	0.2005	0.2436	0.4136	0.1911
		$\vee$	$*$	$*$	$*$	0.2268	0.2591	0.6070	0.2060
		$\wedge$	$*$	$*$	$*$	0.2265	0.2593	**0.6131**	**0.2062**
	$\text {TF}_{\text {BM25}}$	$\vee$	1.2	0.7	$*$	0.2301	0.2573	0.5631	0.2033
		–	$*$	$*$	–	0.1983	0.2597	0.3987	0.1937
		$\vee$	$*$	$*$	$*$	**0.2339**	**0.2718**	0.6028	0.2023
		$\wedge$	$*$	$*$	$*$	0.2010	0.2636	0.4089	0.1926
	$\text {TF}_{\text {constant}}$	–	$>0$	$*$	–	0.0735	0.1868	0.0727	0.0309
		$\vee$	$>0$	$*$	$*$	0.1198	0.2075	0.2645	0.0553
		$\wedge$	$>0$	$*$	$*$	0.0740	0.1881	0.0735	0.0291

**Table 10:** Comparison of the fivefold cross validation of the trained D-LM and TF-$\text {IDF}_{\text {L}}$ models candidates observed in Tables 7 and 8

Challenge	P	C	D-LM	TF-$\text {IDF}_{\text {L}}$
HARD’05	Non-elite	$\vee$	**0.2288**	**0.1523**
		$\wedge$	0.1998	0.0967
	Elite	$\vee$	0.2258	0.1369
		$\wedge$	0.1912	0.0753
Ad Hoc 8	Non-elite	$\vee$	**0.2679**	**0.1600**
		$\wedge$	0.2539	0.1500
	Elite	$\vee$	0.2653	0.0821
		$\wedge$	0.2556	0.0688
eHealth’14	Non-elite	$\vee$	0.5740	**0.4545**
		$\wedge$	0.4060	0.1623
	Elite	$\vee$	**0.5769**	0.4116
		$\wedge$	0.3927	0.1231
Web’02	Non-elite	$\vee$	0.2051	**0.0450**
		$\wedge$	0.2011	0.0250
	Elite	$\vee$	**0.2092**	0.0393
		$\wedge$	0.2010	0.0183

For each test collections: HARD 2005 in Table 3, Ad Hoc 8 in Table 4, eHealth 2014 in Table 5, and Web 2002 in Table 6, we present the results obtained with the TF-IDF model variants and the two pivotizations. In these tables we observe each model with either its standard configuration (S), or its trained configuration (T), obtained taking the configuration that maximizes the evaluation measure $\text {AP}$. The standard parameters of the normalizations for the TF quantifications: $\text {TF}_{\text {total}}$, $\text {TF}_{\text {log}}$ and $\text {TF}_{\text {constant}}$, have the effect of disabling the normalization component ($b=0$). However, for $\text {TF}_{\text {BM25}}$ this does not happen. Thereby, we can study the effect of the parameter *a* in its standard parametrization. To do this we extract the best value obtained with the standard $k_1$ and *b* by selecting the maximum value of the measure $\text {AP}$ obtained by varying the parameter *a*. In case of the trained parameter values instead, for all the ${\text {TF}}$ quantifications, we show in the first row the best result obtained maximizing the $\text {AP}$ without the use of verboseness in the scoring function ($a=1$), and then we show the result obtained when verboseness is added in the scoring function. The tables distinguish between the conjunctive ($\wedge$) and disjunctive ($\vee$) combinations of document verboseness and length.

$\text {TF}_{\text {BM25}}$ works generally better than the other ${\text {TF}}$ quantifications, but not for all test collections. For the test collection eHealth 2014 $\text {TF}_{\text {log}}$ is better.

We also observe that best configuration is achieved using the elite pivotization. The conjunctive combination works generally better than the disjunctive case (24 of 32 experiments better than the disjunctive, all 7 unfavorable cases occur when using the Web 2002 test collection).

In Table 7, we present the results obtained for every test collections using D-LM with $\lambda _{d}$ extended with verboseness. For this model the standard parameter is when $b=1$, and $a=0$, which reduces the formula to the standard D-LM without verboseness (citealtZhai:2001:SSM:383952.384019). This variant is shown on the first row for every test collection. The subsequent rows present the variant of $\lambda _{d}$ when combined with verboseness in disjunction and conjunction with non-elite and elite pivots. For this model we observe that the presence of verboseness produces for only one test collection significant improvements. Overall we observe that the non-elite pivotization should be preferred (all the experiments produce better results than the elite one). No difference is observed by using a disjunctive or conjunctive combination of the pivots.

In Table 8, we present the results obtained for every test collections using TF-$\text {IDF}_\text {L}$ model with $\lambda _{q}$ that combines in a LM fashion the term length and burstiness. For this model the standard parameter is when $\lambda _q = 1$, which reduces this IR model to a non TF-normalized $\text {TF}_{\text {total}}$-IDF model. This variant is shown on the first row for every test collection. The following rows present the variant of $\lambda _{q}$ when combined in disjunction and conjunction with non-elite and elite pivots. We observe that this parametrization produces significantly better results than the standard case, and that the non-elite parametrization should be preferred. Also here, as for D-LM, no difference is observed by using a disjunctive or conjunctive combination of the pivots. We also observe that overall the values of the trained parameter *a* is often equal to 1, which suggests that, for these model variants, the term length does not play an important role in adjusting the document’s score. This is a curious behavior since it is dual to the D-LM model, where the document verboseness does not play an important role either.

Finally, in Tables 9 and 10 we present the results of the fivefold cross validation for all the trained cases of the TF-IDF models, in the first table, and the D-LM and TF-$\text {IDF}_\text {L}$ models, in the second table.

**Fig. 4:**
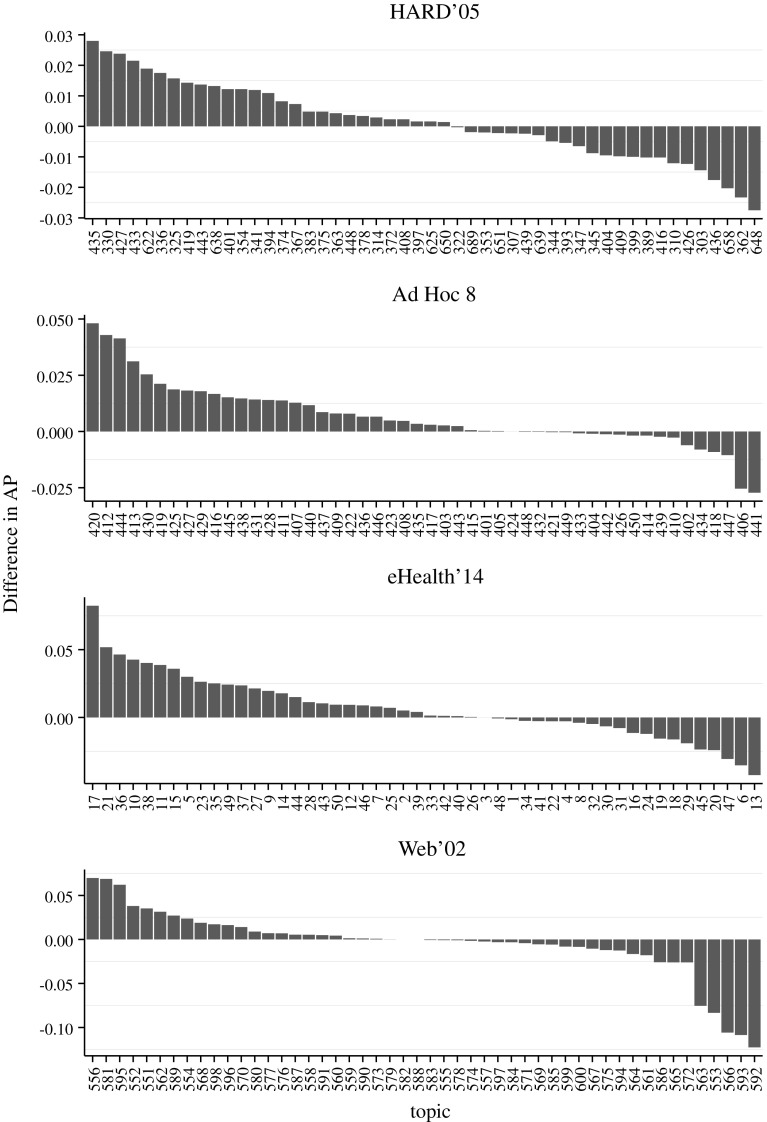
Difference on a per topic based between the $\text {AP}$ of the trained $\text {TF}_{\text {BM25}}$-IDF with verboseness combined in conjunction with elite pivots, and the trained classic $\text {TF}_{\text {BM25}}$-IDF. When the difference is positive the variant with verboseness performs better than the classic version

## Analysis and discussion

Finally we make some observations across the experimental results about the behavior of the parameter *a*. Before that however, let us make an observation on the nature of the data at our disposal. Figure 3 shows the distribution of the document verboseness versus document length for the elite and non-elite pivotizations. In both cases we see that verboseness brings additional information compared to document length: the plotted distributions are well spread, away from the first diagonal.

Comparing the two distributions, it is interesting to observe that the non-elite pivotization is significantly more skewed than the elite one: the x-axis of the left plot has a scale in the (0, 0.02) range, while the one on the right plot has a scale that matches the y-scale: (0, 4). This supports and grounds our hypothesis that elite pivotization should provide us better means to balance verboseness and document length with parameter *a*.

The *a* parameter controls the contribution of elite pivoted verboseness and elite pivoted document length. When $a<0.5$, the contribution of the document verboseness is higher than the contribution of the document length, and vice versa when $a>0.5$. Looking at the distribution for the elite pivotizations of the documents, redefining the origin to the point (1, 1) we split the distributions in four quadrants.^4^ We know that whatever *a* we fix, the documents in the I quadrant will be always demoted to some degree, and in the III quadrant the documents will be always promoted to some degree. So here the question is what happens to the documents in the IV and II quadrant. When to be preferred is the contribution of document verboseness ($a>0.5$) more documents with low verboseness ($\hat{v}_d<1$) and high length ($\hat{l}_d>1$) will be promoted against the documents of the IV quadrant, and when preferred is the contribution of the document length ($a<0.5$) the contrary happens. Therefore, the *a* values, previously listed, should anti-correlate with the ratio of the number of relevant documents between the II quadrant and the IV quadrant. Here the two lists of values sorted by test collection, of *a* extracted from Tables 3, 4, 5, and 6, for the standard BM25 case with trained *a*: 0.8, 0.6, 0.4, and 0.0 and ratios: 0.63, 0.86, 1.16 and 4.20, where we observe that they anti-correlate. Therefore if we think that all the documents of the collection should be relevant we should find the *a* value that mostly balance the proportion of non verbose but long documents with the short but verbose documents. All the test collections but Disks 4&5 have been crawled from the Web. For all of them we can observe that the plots manifest a visible noise. In particular we observe the presence of black dots that are most probably caused by the existance of duplicated documents in the collections. For example, the existance of duplicated documents in the e-Health’14 test collection is a known issue to the e-Health IR community.

In Tables 3, 4, 5, and 6 we observe that the best performing configuration, for both $\text {TF}_{\text {log}}$ and $\text {TF}_{\text {total}}$, uses the trained parameters combined in disjunction, in particular in Table 4 these configurations also show statistical significance against both standard configuration and trained configuration when verboseness is not present ($a=0$). The elite pivotization performs generally better than the non-elite pivotization. In particular the best performing configurations are with elite pivotization and trained parameters in conjunction. We observe also that in general the elite pivotization weighting role is taken by the parameter *a* ($b=1$ means that a full document verboseness and length normalization is applied).

In Fig. 4 we further analyze the best configuration on a per topic basis. Here, we show the difference in $\text {AP}$ between the $\text {AP}$ of the trained TF$_\text {BM25}$-IDF with verboseness combined in conjunction with elite pivots, and the trained classic TF$_\text {BM25}$-IDF. If the difference is positive the variant with verboseness is better than the classic version.

## Conclusion

This paper presents an extensive study of ${\text {TF}}$ quantifications and normalizations. The quantifications are with respect to a well-defined spectrum comprising $\text {TF}_{\text {total}}$, $\text {TF}_{\text {log}}$, $\text {TF}_{\text {BM25}}$, and $\text {TF}_{\text {constant}}$. Each of these ${\text {TF}}$ quantifications reflects a dependence assumption. In particular, $\text {TF}_{\text {total}}$ and $\text {TF}_{\text {constant}}$ are the extremes of the quantification spectrum, assuming independence for the former and subsumption for the latter. $\text {TF}_{\text {BM25}}$ is a relatively strong dependence assumption, and $\text {TF}_{\text {log}}$ is in the middle between $\text {TF}_{\text {total}}$ and $\text {TF}_{\text {BM25}}$. Each of these quantifications incorporates a ${\text {TF}}$ *normalization* parameter, usually denoted as $K_d$.

Whereas current approaches regarding $K_d$ consider only the document length as parameter of $K_d$, this paper makes the case for $K_d$ to be a combination of *document verboseness and length*. There are many heuristic options for how to combine the parameters, and this paper contributes the theoretical foundations leading to a systematic combination of document verboseness and length.

The paper reports results of an experimental study investigating the effect of various settings of $K_d$ for the four main ${\text {TF}}$ quantifications. The overall finding is that combining document verboseness with document length (either in a conjunctive or disjunctive way) improves retrieval quality when compared to results considering document length only.

We expand this in two directions, first by exploring a similar normalization in the context of LM and second a similar normalization in the context of TF-IDF. For the former, we include document verboseness into the Dirichlet smoothing where non-significant effect is observed, which signifies that document verboseness can be neglected. For the latter, in Sect. 4.3 we have observed the duality between document verboseness and document length on one side, and term burstiness and term length on the other side, and we observed the effect of these normalizations on the query side with respect to LM. Here, significant improvements are observed, however these improvements are obtained primarily by the use of term burstiness, while the term length can be neglected. In both directions improvements are observed given by the new parametrizations, and their results show a dual behavior, given by the exclusion of document verboseness in the former, and by the exclusion of term length in the latter.

In summary in this paper we have provided an exhaustive study of normalization factors in IR probabilistic models using 4 different test collections. Based on the observations made on these test collections, we have made the case that different domains, having different text statistics, can be directly factored into the existing probabilistic models. We have thus provided a quantification of the various document and term statistics into one factor that balances different prior probabilities that all these models, more or less explicitly, rely on.

## Footnotes

1 Although the “length of a term” is non intuitive, here it is meant the L1-length of a vector

2 Software available on the website of the first author.

3 http://www.terrier.org.

4 Here we enumerate the quadrants from the top right, I and then going counter-clockwise the others: II, III and IV.
